# Integrating Machine Learning and SHAP Analysis to Advance the Rational Design of Benzothiadiazole Derivatives with Tailored Photophysical Properties

**DOI:** 10.1021/acs.jcim.4c02414

**Published:** 2025-04-29

**Authors:** Rafael F. Veríssimo, Pedro H. F. Matias, Mateus R. Barbosa, Flávio O. S. Neto, Brenno A. D. Neto, Heibbe C. B. de Oliveira

**Affiliations:** † Laboratório de Estrutura Eletrônica e Dinâmica Molecular, 67824 Universidade Federal de Goiás, 74690-900 Goiânia, GO, Brasil; ‡ Instituto Federal de Educação, Ciência e Tecnologia de Goiás, 72876-601 Valparaıso de Goiás, GO, Brasil; § Laboratório de Quımica Medicinal e Tecnológica, Institute of Chemistry, 28127 Universidade de Brasılia, 70910-900 Brasılia, DF, Brasil

## Abstract

2,1,3-Benzothiadiazole (BTD) derivatives show promise in advanced photophysical applications, but designing molecules with optimal desired properties remains challenging due to complex structure–property relationships. Existing computational methods have a high cost when predicting precise photophysical characteristics. Machine learning with Morgan fingerprints was employed to forecast BTD derivative maximum absorption and emission wavelengths. Three flavors of machine learning models were applied, namely, Random Forest, LigthGBM, and XGBoost. Random forest achieved *R* ^2^ values of 0.92 for absorption and 0.89 for emission, validated internally with 10-fold cross-validations and externally with recent experimental data. SHapley Additive exPlanations (SHAP) analysis revealed critical design insights, highlighting the tertiary amine presence and solvent polarity as key drivers of red-shifted emissions. By the development of a web-based predictive tool, the potential of machine learning to accelerate molecular design is demonstrated, providing researchers a powerful approach to engineer BTD derivatives with enhanced photophysical properties.

## Graphical abstract

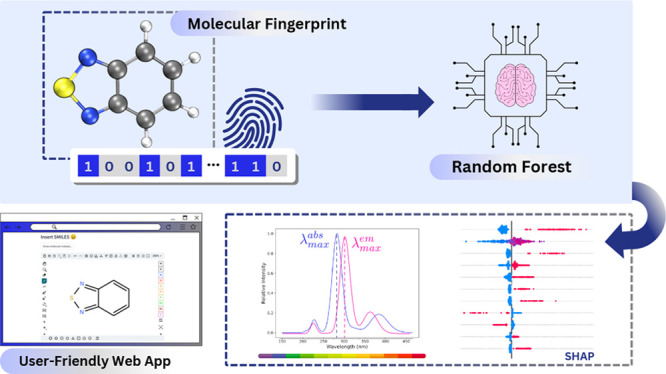

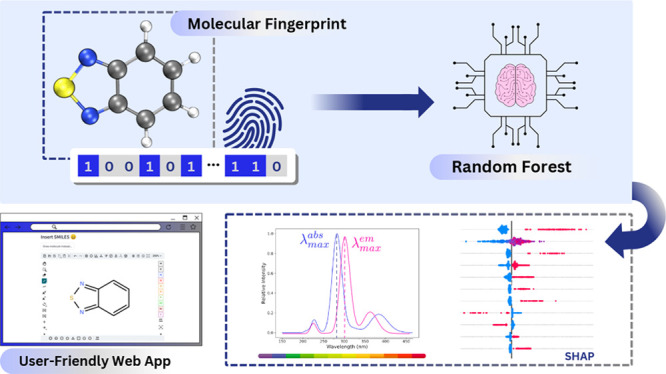

## Introduction

2,1,3-Benzothiadiazole (BTD) derivatives have attracted significant attention due to their versatile photophysical properties and potential applications across various scientific and technological domains. These heterocyclic systems, featuring a fused thiadiazole ring and strong electron-withdrawing capacity,^1–3^ have been widely explored in organic photovoltaics,^4^ optoelectronics,^5^ and as fluorescent probes in bioimaging,^6,7^ among other applications (Figure 1). BTD-based fluorophores, known for their brightness,^8^ large Stokes shifts,^9,10^ and strong intramolecular charge transfer (ICT) interactions,^11–13^ have demonstrated remarkable utility in fields where precise photophysical behavior is paramount. These derivatives often form ordered crystalline packings and can be tuned structurally to achieve the desired optical outputs. Such adaptability has led to their incorporation in devices requiring finely controlled emission spectra,^14^ as well as their application in advanced fluorescence imaging, including the selective staining of mitochondria in cancer cell lines.^15–17^

**Figure 1:**
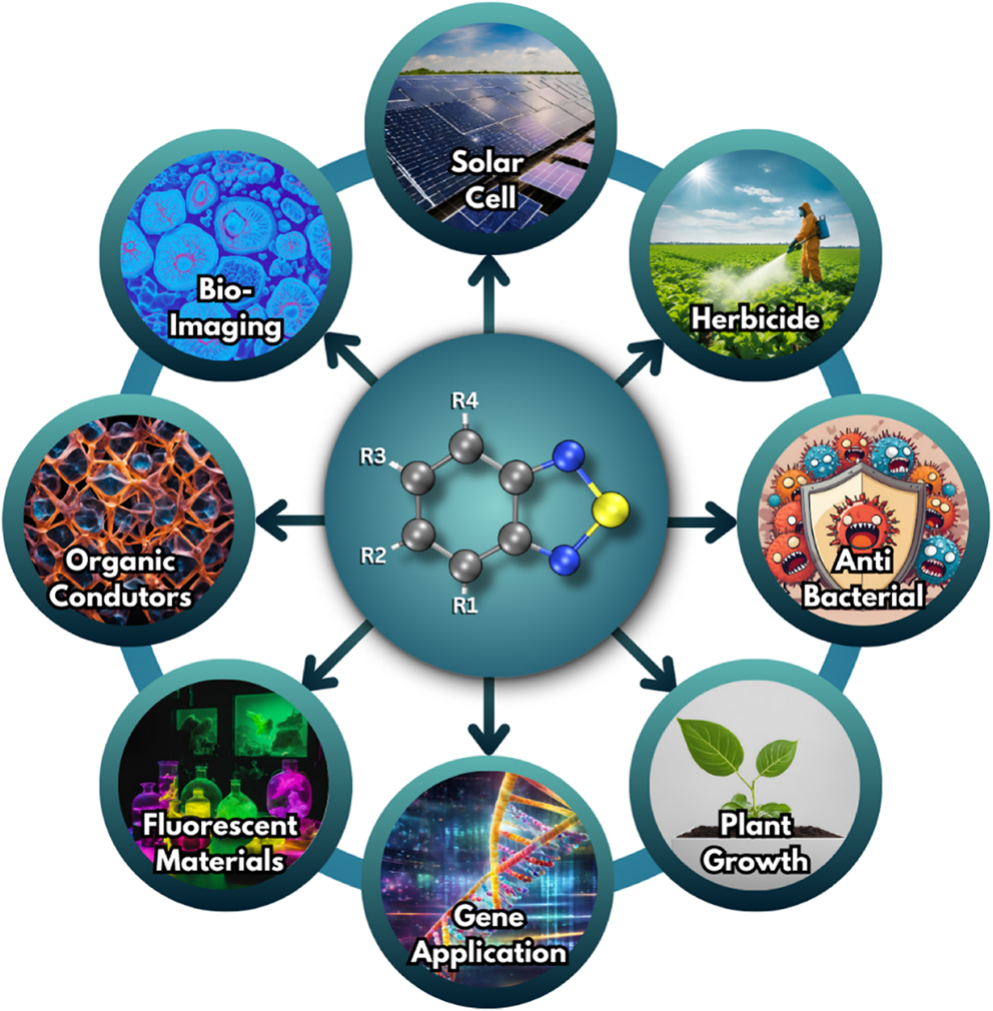
Schematic representing the versatile applications of benzothiadiazole in various fields, including solar cells, bioimaging, herbicides, and more, showcasing its potential in advancing technology and science.

Despite the promise of BTD analogues, identifying optimal candidates with target photophysical characteristics frequently relies on iterative synthetic efforts, extensive experimental screenings, or high-level computational chemistry. Traditional quantum chemical protocols, although insightful, can be expensive and time-consuming,^14,15^ limiting their routine use for rapid compound discovery. This challenge is compounded by the subtle interplay of substituents, solvent polarity, and molecular conformation on the electronic transitions governing absorption and emission.^18^ Consequently, there is a need for more accessible, efficient, and systematic strategies to predict photophysical properties without relying exclusively on expensive or specialized computational tools.

In recent years, machine learning (ML) methodologies have emerged as powerful alternatives for predicting molecular properties with reduced computational demands.^19^ By leveraging large data sets and pattern recognition capabilities, ML models can correlate structural features with observed properties, bypassing the necessity for explicit quantum chemical descriptor calculations.^20,21^ A particularly successful approach involves representing molecules through molecular fingerprints (e.g., Morgan or MACCS), which encode the presence or absence of specific substructures as binary patterns.^22^ These fingerprints provide a straightforward yet effective means of capturing chemical information relevant to photophysical phenomena.

Recent studies have shown that ML models combined with molecular fingerprints can predict various chemical properties, including kinetic parameters and other physicochemical traits, with accuracy comparable to or even surpassing conventional QSAR/QSPR methods.^20,23^ Recently, Ju et al. compiled a database of more than 3000 distinct organic fluorescent dyes and employed ML models that achieved mean absolute errors as low as 0.13 for quantum yields and 0.080 eV for emission energies, providing a fast and accurate alternative to quantum mechanical methods.^24^ After that, Mai et al. introduced an interpretable ML strategy for azo dyes, pinpointing structural factors such as the number of sulfur atoms and certain C–N topological patterns as key contributors to red shifts in the maximum absorption wavelength, and successfully identified 26 new azo molecules with enhanced λ_max_ values through high-throughput screening.^25^ Lately, Mahato and Kumar applied optimized ML techniques to predict absorption and emission wavelengths, as well as quantum yields for 3066 organic dyes, obtaining *R* ^2^ values as high as 0.961 for absorption wavelengths, thereby surpassing previous state-of-the-art benchmarks and allowing more efficient discovery of high-performance dyes.^21^

The implications of such ML-driven approaches for BTD compounds are substantial. Beyond accelerating the design of novel compounds with tailored absorption and emission properties. Interpretable ML techniques (such as SHapley Additive exPlanations, SHAP) can shed light on how specific structural motifs influence photophysical behavior. This mechanistic understanding is invaluable as it enables researchers to rationalize structure–property relationships and to guide synthetic chemists in selecting substituents or solvents that yield the desired optical responses. Moreover, the development of user-friendly online platforms can broaden access to these predictive tools for nonspecialists, facilitating cross-disciplinary collaborations and expediting advancements in photophysical material design.

The goal of this study is to develop a predictive protocol capable of accurately estimating the photophysical properties of BTD derivatives by combining expertises in BTD chemistry,^26,27^ quantum mechanical calculations,^28–30^ and machine learning workflows.^20,31^ By systematically correlating molecular fingerprints with absorption and emission wavelengths, we identify key structural motifs and substituents that govern these optical behaviors. This approach enables the rational design of novel BTD-based materials with tailored photophysical properties, leveraging an integrated and interpretable ML-driven framework to optimize the performance and guide future experimental efforts.

## Methodology

### Database and Molecular Descriptors

A data set comprising 701 BTD derivatives was assembled to develop machine learning models for predicting their maximum absorption (λ_max_^abs^) and emission (λ_max_^em^) wavelengths. The data were obtained from the Elsevier database using the API key in conjunction with web scraping techniques implemented in a Python 3.x environment. This process involved collecting experimental values reported in the literature, including λ_max_^abs^ and λ_max_^em^, along with the solvents used in the measurements.

For each solvent, the respective normalized molar electronic transition energies (ETN) were collected from the literature^32^ and incorporated in the database. This property is a measure of the solvent’s polarity and is difined by^33^

1 $$E_{T}^{N}=\frac{E_{T}\left(\mathrm{solvent}\right)-E_{T}\left(\mathrm{TMS}\right)}{E_{T}\left(\mathrm{water}\right)-E_{T}\left(\mathrm{TMS}\right)}=\frac{E_{T}\left(\mathrm{solvent}\right)-30.7}{32.4}$$

where *E_T_* (solvent), *E_T_* (TMS), and *E_T_* (water) are the electronic transition energies for the solvent, tetramethylsilane (TMS), and water, respectively. The *E*_T_^*N*^ constant ranges from 0 (for TMS, the least polar solvent) to 1 (for water, the most polar solvent). It is noteworthy that our data set exhibited an imbalance in the distribution of solvents used, which may have introduced biases in the results. This imbalance may affect the generalizability of our findings, especially in predicting molecular properties in diverse solvent environments. Furthermore, variations in solvent polarity, hydrogen bonding capabilities, and normalized molar electronic transition energies could contribute to inconsistencies in the model performance.

To numerically represent the molecular structures, each BTD compound was associated with its simplified molecular input line entry system (SMILES).^34^ These SMILES were converted into circular descriptors using RDKit.^35^ Morgan fingerprints were generated as binary vectors to represent the presence or absence of specific substructures within a molecule.^36^ A scan of the fingerprint length and radius was performed with 512, 1024, 2048, 3072, 4096, and 8192 bits and 1 through 6 radii to determine the optimal representation for model development; these results can be found in the Supporting Information.

### Models and Validation

In light of the recent success of decision tree-based machine learning algorithms applied in predictive chemistry,^37–41^ this study selected three decision tree models, eXtreme Gradient Boosting (XGBoost),^42^ Random Forest (RF),^43^ and light gradient boosting machine (LightGBM).^44^

Random Forest is an ensemble learning method that constructs multiple decision trees during training and outputs the mean prediction of the individual trees.^45^ It mitigates overfitting by averaging the results, leading to an improved predictive accuracy. XGBoost is a scalable, end-to-end tree boosting system that uses a gradient boosting framework and advanced regularization techniques to reduce the loss function and enhance model performance.^46,47^ LightGBM is designed for efficiency with large data sets, offering faster training speeds and higher efficiency compared to traditional boosting algorithms.^48^

The models were developed using the scikit-learn,^49^ XGBoost, and LightGBM Python libraries. Hyperparameter optimization was conducted using the Optuna^50^ python algorithm to fine-tune model parameters. Detailed hyperparameter settings for each algorithm are provided in Table S1 of the Supporting Information.

To compare and estimate performance of the chosen algorithms, coefficient of determination *R* ^2^, explained variance *Q* ^2^, mean absolute error (MAE), and root mean squared error (RMSE) were selected. A 10-fold cross-validation was performed using K-Fold,^51^ the data set was divided equally into subsets to maintain consistency, with nine subsets (630 samples) used for training sets and one subset (70 samples) reserved for testing sets at any given time. This process was repeated 10 times, with the final result being the mean of the outcomes from all ten tests.

External testing was performed using recent experimental data from 20 BTD derivatives not included in the initial data set. These new data points provided an independent test to evaluate the predictive capability of the models for unseen compounds. The predicted λ_max_^abs^ and λ_max_^em^ values were compared with the experimental values, and the relative errors were calculated for each molecule to assess prediction accuracy.

To further assess the robustness of the models and ensure that their predictive performance was not due to chance correlations, we performed y-randomization tests. In this procedure, the dependent variable values were randomly shuffled while keeping the independent variables unchanged.^52,53^

Residual plots were also analyzed as part of the validation process. These plots display the differences between experimental and predicted values against predicted values. A random scatter of residuals around zero indicates that the model assumptions are valid and that the model provides an appropriate fit to the data.

### Model Interpretation and Deployment

An essential requirement in developing predictive models is the mechanistic interpretation of the predictions of the model.^54^ Understanding how the model makes its predictions provides valuable chemical insights and improves confidence in its reliability.^55^ To achieve this, SHapley Additive exPlanations (SHAP) was employed as a unified approach for interpreting model predictions.^56^

The algorithm was derived from game theory to estimate the importance of each player in a cooperative game.^57^ A reward is assigned to each player (substructure) based on their contribution to the result of their games (properties). The SHAP value Φ_*i*_ for the *i* feature is calculated as follows

2 $$\Phi_{i}=\frac{1}{\left|N\right|!}\sum_{S\subseteq N\backslash \left\{i\right\}}\frac{\left|S\right|!\left(\left|N\right|-\left|S\right|-1\right)!}{\left(\left|N\right|-1\right)!}\left[f\left(S\cup \left\{i\right\}\right)-f\left(S\right)\right]$$

where *S* represents a subset of features and *N* is the complete set of all features. It is computed as the average marginal contribution of feature *i* across all possible permutations of the feature set. This involves evaluating the change in the model’s output when feature *i* is added independently to the subsets *S*. The significance of the feature is reflected in its impact on the variation of the model’s output. A schematic representation of the workflow is provided in Figure 2.

**Figure 2:**
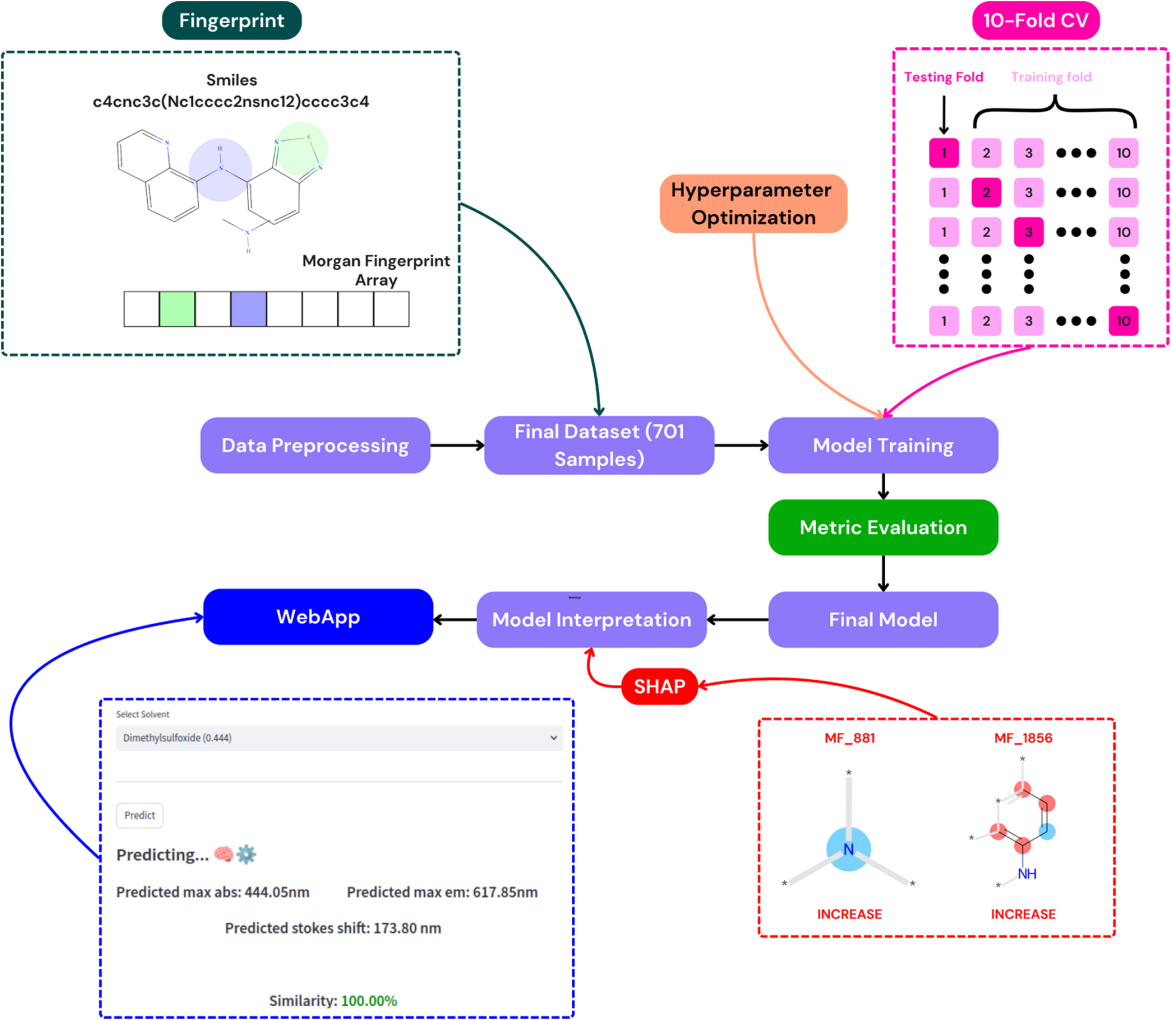
Schematic representation of the model development workflow, illustrating key steps, including data collection, preprocessing, fingerprint generation, hyperparameter optimization using Optuna, 10-fold cross-validation, model training with 3 algorithms (eXtreme Gradient Boosting, Random Forest, Light Gradient Boosting Machine), metric evaluation (*R* ^2^, *Q* ^2^, MAE, RMSE), SHAP-based model interpretation, and deployment as a web application for prediction and visualization of results.

Using Morgan fingerprints as molecular descriptors, SHAP values provide a solution to assign a quantitative measure of importance to each molecular substructure in predicting maximum absorption (λ_max_^abs^) and emission (λ_max_^em^) wavelengths. This approach connects the need to interpret the model’s predictions with understanding the photophysical properties of BTD derivatives, as it helps identify which molecular features most significantly influence these properties.

The web application was deployed on a cloud-based platform utilizing the Streamlit environment to provide a responsive and user-friendly interface. Machine learning predictions are integrated into the workflow, allowing the rapid evaluation of BTD derivatives. The application incorporates the Ketcher molecular editor, an open-source web-based tool for drawing and editing chemical structures, enhancing both the user experience and data accuracy. Prediction results are displayed on the screen for immediate review and can also be exported as spreadsheets for further analysis.

## Results and Discussion

### Validations

Four statistical criteria, coefficient of determination (*R* ^2^), explained variance (*Q* ^2^), mean absolute error (MAE), and root mean squared error (RMSE) were employed to assess the internal validation of the models via 10-fold cross-validation. The average values for each metric are listed in Table 1. Notably, the standard deviations for MAE and RMSE are expressed in nanometers (nm), facilitating direct interpretation in the context of absorption and emission wavelengths.

**Table 1:** Performance Comparison of Three Machine Learning Algorithms XGBosst, Randon Forest, and LigthGBM in Predicting Photophysical Properties (λ_max_^abs^ and λ_max_^em^) Using a 10-Fold Cross-Validation^a^

photophysical property	algorithm	*R* ^2[*a*]^	*Q* ^2[*a*]^	MAE (nm)	RMSE (nm)
max absorption	XGBoost	0.8976 ± 0.1075	0.8999 ± 0.1039	7.2155 ± 1.2992	14.9539 ± 8.4224
	Randon Forest	0.9252 ± 0.0400	0.9259 ± 0.0401	6.8863 ± 0.8979	12.0208 ± 2.8983
	LightGBM	0.8981 ± 0.0377	0.8993 ± 0.0373	8.0827 ± 1.3499	14.3505 ± 3.6646
max emission	XGBoost	0.8909 ± 0.1054	0.8937 ± 0.1023	13.3673 ± 2.6616	21.2640 ± 9.9791
	Randon Forest	0.8884 ± 0.0300	0.8923 ± 0.0280	14.8029 ± 1.1918	21.4308 ± 1.7766
	LightGBM	0.8740 ± 0.0508	0.8763 ± 0.0488	14.5597 ± 1.8141	21.7825 ± 3.2040

a For each property, the metrics *R* ^2^, *Q* ^2^, MAE, and RMSE are reported along with their standard deviations.

Among the tested models, Random Forest provided the most reliable predictions for λ_max_^abs^, achieving an *R* ^2^ of 0.9252 with comparatively lower MAE and smaller standard deviations. While XGBoost performed better for λ_max_^em^ with *R* ^2^ = 0.8909, its larger standard deviations and higher *Q* ^2^ values suggest less consistency. Given that the *R* ^2^ values for Random Forest and XGBoost are relatively close, the more stable performance of Random Forest led to its selection for all subsequent discussions. In essence, the model offering accurate results and reliable predictive stability was favored.

All models produced similar *R* ^2^ and *Q* ^2^ values, indicating a strong goodness-of-fit and low likelihood of overfitting. The observed differences, generally confined to the second or third decimal place and below 0.2, reaffirm the models’ robustness.^53^ To further confirm that the models were not merely memorizing the training data, a y-randomization test was performed. This involved retraining the model with randomly shuffled target values, resulting in significantly poorer *R* ^2^ and *Q* ^2^ metrics. Such a drop in the performance upon randomization confirms that the original models captured meaningful structure–property relationships rather than spurious correlations. The complete details and results of the *y*-randomization procedure are provided in the Supporting Information (Figure S1).

Although these internal validation results are promising, internal metrics alone cannot guarantee the model’s reliability in real-world scenarios. To address this, an external testing was performed using recently published experimental data. Despite making up less than 3% of the original database, the external set introduces newly published and structurally diverse molecules, providing a valuable and independent assessment of the model’s ability to generalize beyond its training domain. By comparing predicted values to newly reported experimental results, the model’s generalization to unseen compounds was evaluated. The prediction errors, expressed as percentage differences from experimental data, are compiled in Table 2. This step provides practical evidence of the model’s capacity to extend beyond the initial data set.

**Table 2:**
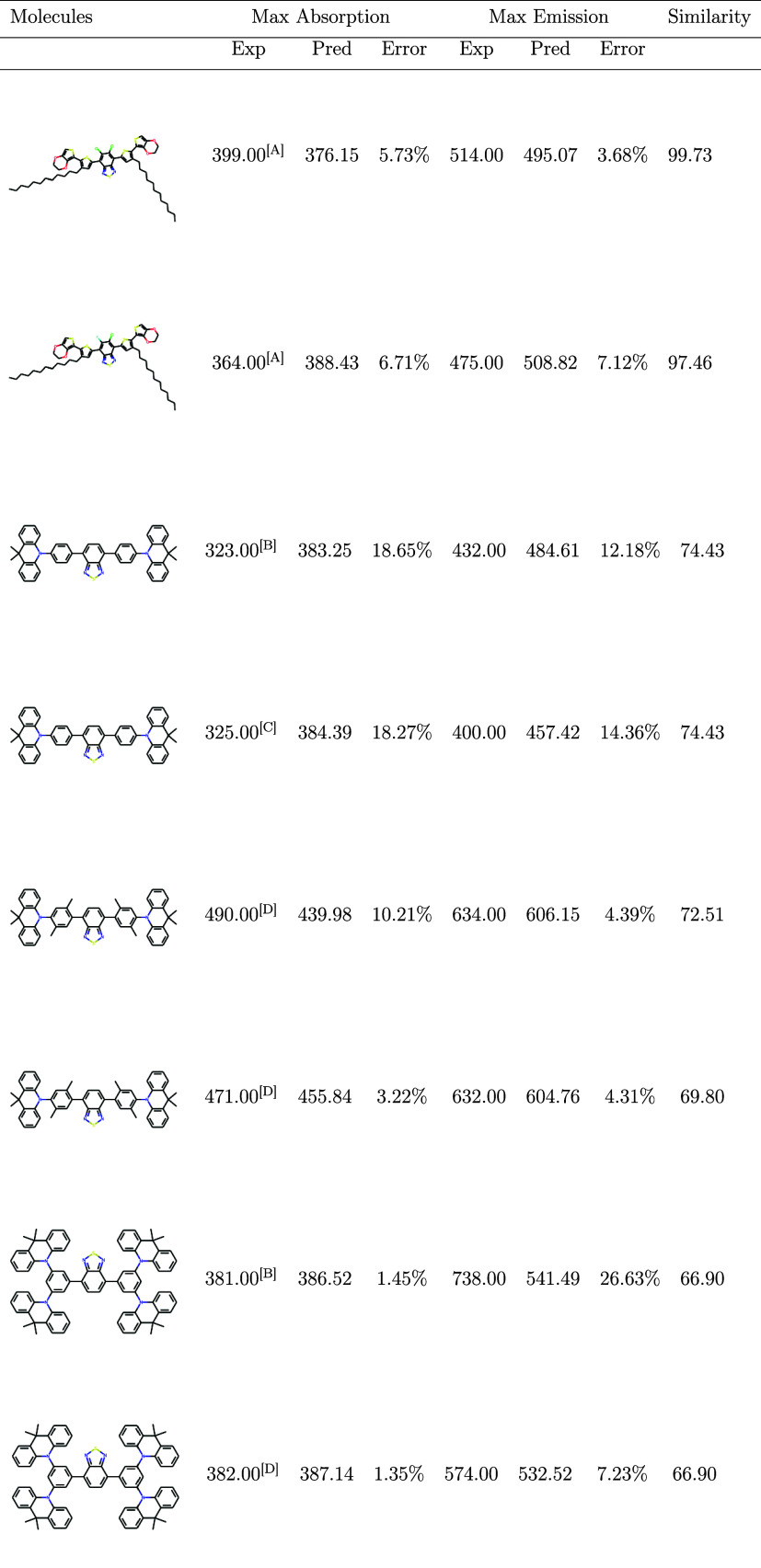

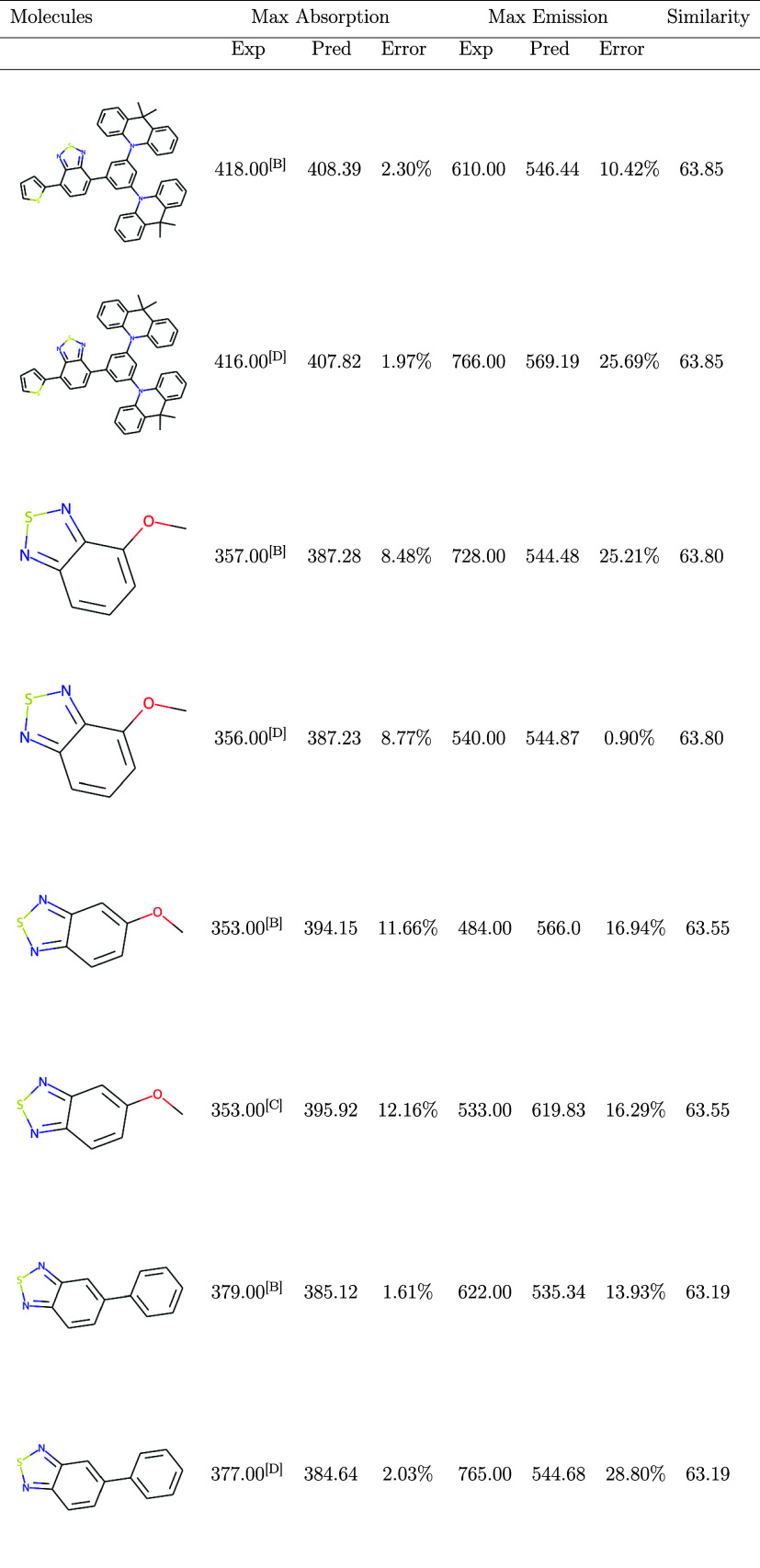

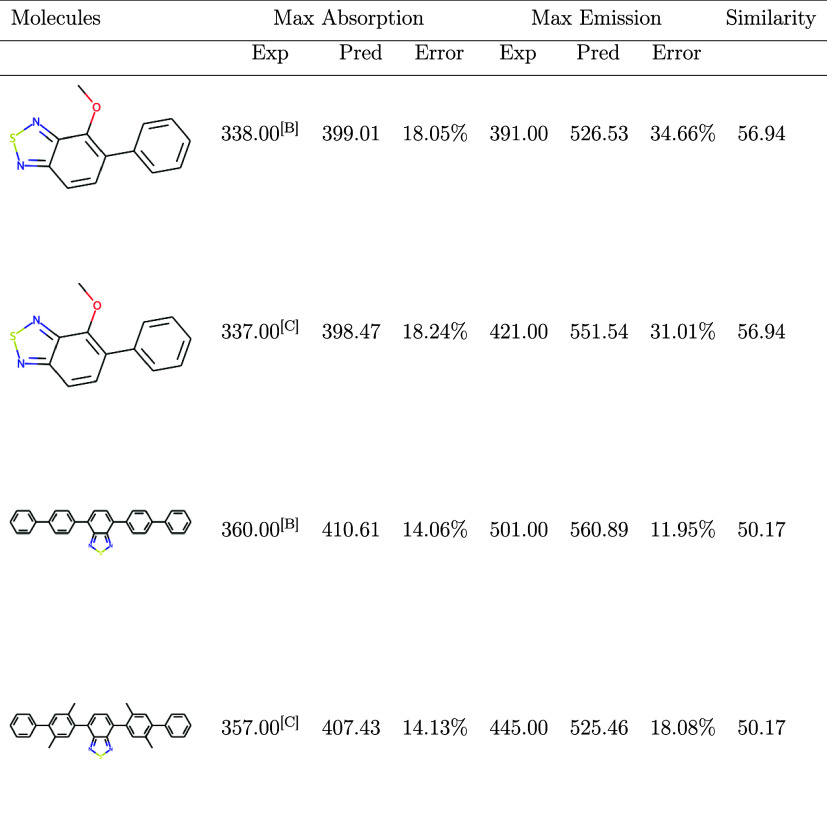
External Testing of the Model in Predicting Maximum Absorption and Maximum Emission with Experimental Data from the Literature in nm^a^

a Experimental values (Exp) were measured in different solvents: [A] tetrahydrofuran (THF), [B] toluene (TOL), [C] methanol (MeOH), and [D] dichloromethane (DCM). Predicted values (Pred) represent the model’s outputs. Also, with the similarity in %.

The analysis of error and similarity reveals a clear trend: molecules with high structural similarity tend to have lower prediction errors, while those with lower similarity show increasing errors. In particular, molecules with very low similarity produce unreliable results, leading to larger errors and indicating that they fall outside the model’s reliable prediction range. However, some low-similarity molecules still exhibit minimal errors, suggesting that similarity alone is not always a perfect predictor of model performance, as certain key features may still align well with the model’s learned patterns. Additionally, the emission properties appear to be slightly more sensitive to structural variations. Nevertheless, caution should be exercised when interpreting predictions for low-similarity molecules, as their accurate results may arise from coincidental correlations rather than a true generalization by the model.

A scatter plot was generated to more clearly visualize the relationship between predicted and experimental values for RF, XGBoost, and LightGBM models (see Figures 3, S3 and S4, respectively). On these plots, the *x*-axis represents experimental (actual) values, while the *y*-axis displays predictions. Ideally, points should align closely along the diagonal line (*r* ^2^ = 1), indicating near-perfect agreement. Significant deviations from this line would suggest inaccurate predictions. As shown in Figure 3, Random Forest predictions cluster closely around the diagonal, reinforcing the conclusion that this model offers a balanced combination of accuracy and consistency for forecasting the photophysical properties of BTD derivatives. Additionally, it is important to note that some of the data used in this study were derived from DFT/TDDFT calculations; since the accuracy of TDDFT predictions for absorption and emission spectra strongly depends on the selected density functional, minor deviations may occur, potentially influencing the overall predictive performance.

**Figure 3:**
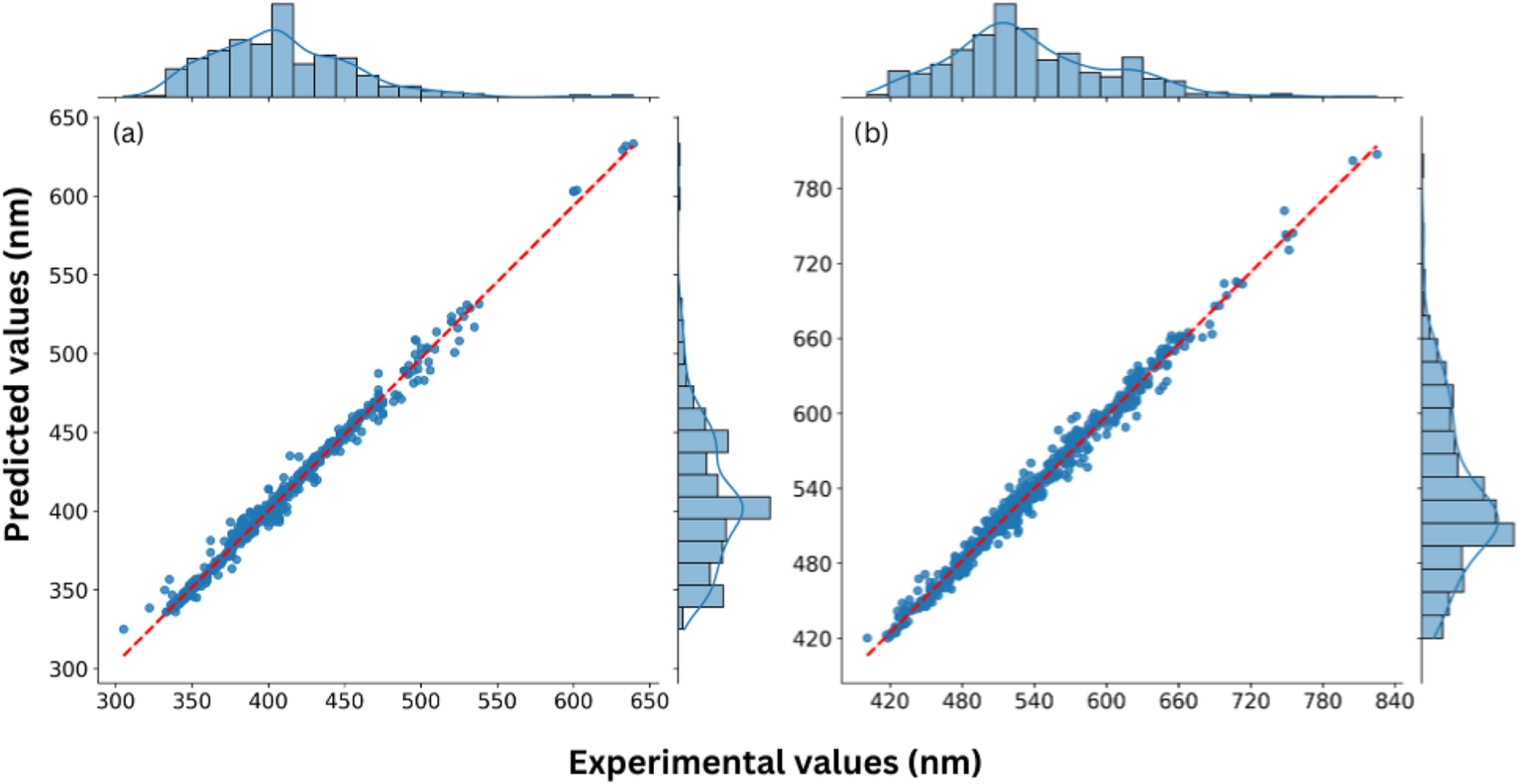
Predicted values versus experimental values for (a) max absorption and (b) max emission for Randon Forest; red line represents the ideal prediction and blue points the actual prediction.

### SHapley Additive exPlanations (SHAP)

Statistical metrics alone cannot fully explain how machine learning models arrive at their predictions. Therefore, SHAP values were employed to determine the features that exert the greatest influence on the predicted properties. SHAP assigns a numerical score to each feature, indicating its relative contribution to the model’s final output.

Figures 4a and 5a present the top 11 most influential features for both the absorption and emission models, respectively, along with their SHAP values. In the case of λ_max_^abs^, no single feature strongly alters the predictions. On the other hand, for λ_max_^em^, the ETN constant and feature 881 (a tertiary amine) exert a more substantial effect on the model’s outputs.

**Figure 4:**
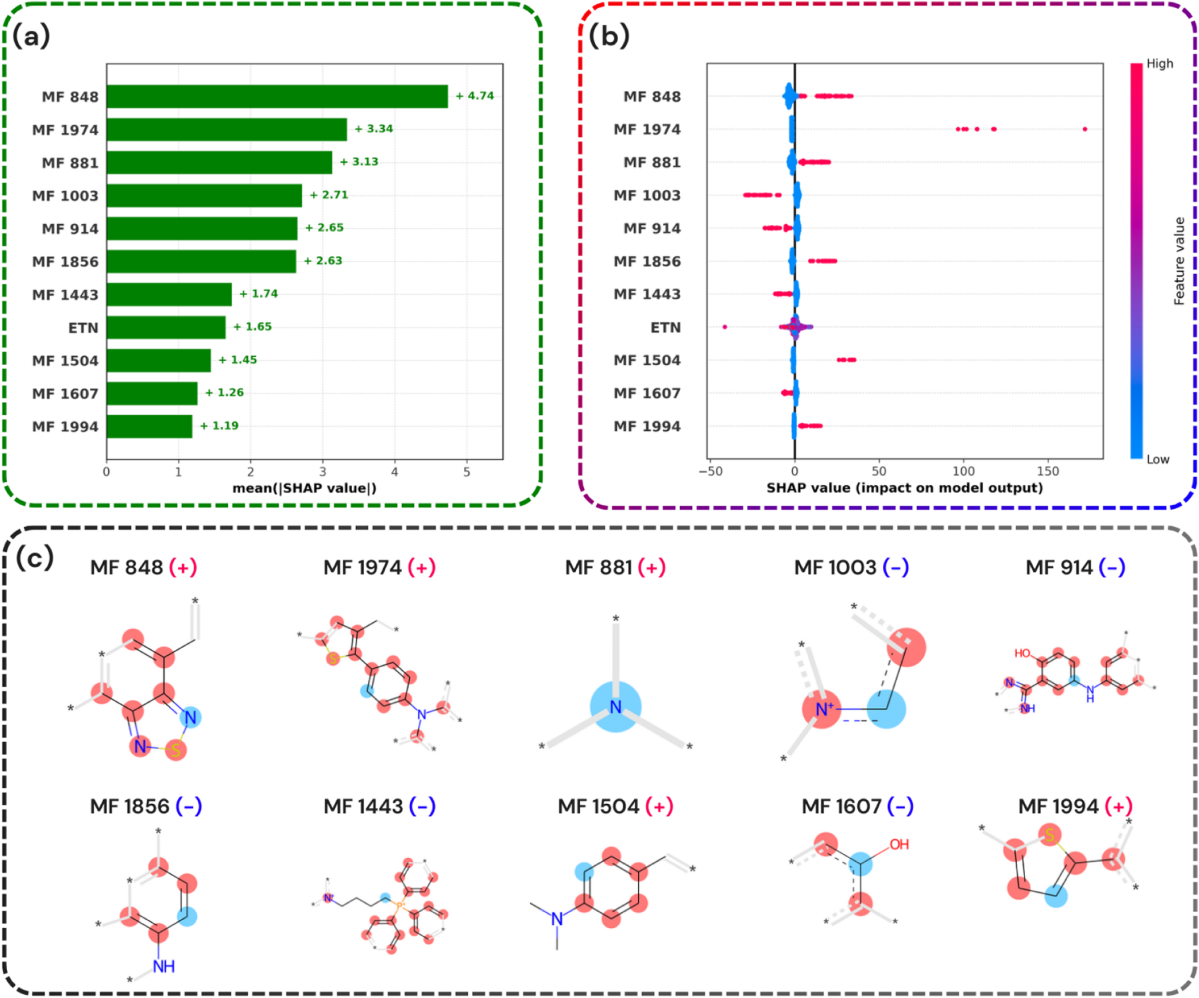
Visualization of SHAP analysis and molecular features influencing the predictive λ_max_^abs^ model. (a) Mean SHAP values of top molecular features (MFs) highlight their overall importance. (b) Bee swarm plot showing SHAP values for each molecular feature with color indicating feature magnitude (high: red, low: blue). (c) Molecular structures of selected features, classified as positively (+) or negatively (−) contributing to the model’s output.

**Figure 5:**
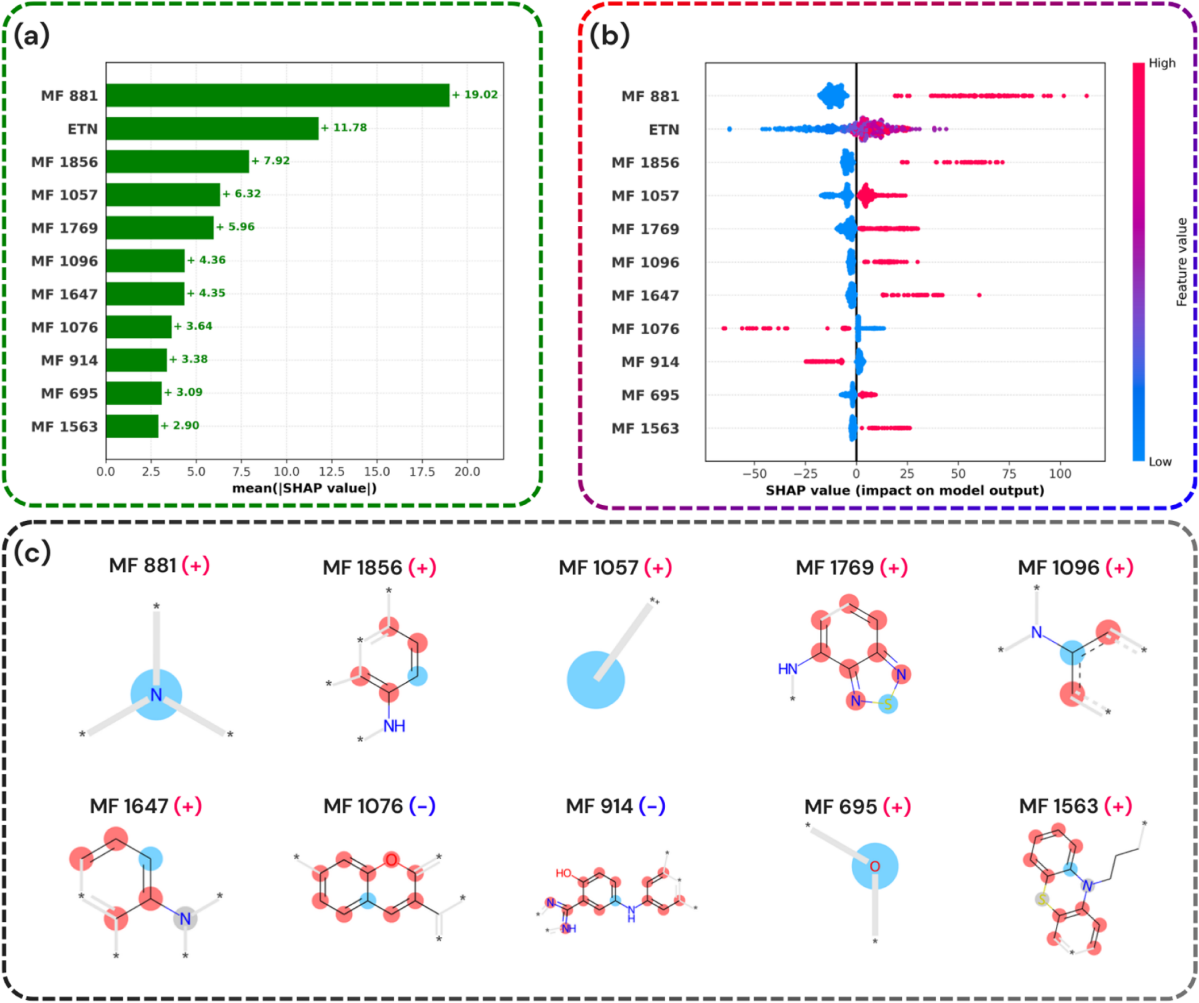
Visualization of SHAP analysis and molecular features influencing the predictive λ_max_^em^ model. (a) Mean SHAP values of top molecular features (MFs) highlight their overall importance. (b) Bee swarm plot showing SHAP values for each molecular feature with color indicating feature magnitude (high: red, low: blue). (c) Molecular structures of selected features, classified as positively (+) or negatively (−) contributing to the model’s output.

In Figures 4b and 5b, red indicates the presence of a particular feature, while blue denotes its absence. The *x*-axis represents the SHAP values, illustrating whether the presence or absence of a given substructure increases or decreases the predicted λ_max_^abs^ or λ_max_^em^. For instance, feature 881 has a positive SHAP value, meaning that its presence elevates the predicted maximum absorption.

As shown in Figures 4a and 5a, listing the top substructures for absorption and emission (with their effects visualized in Figures 4c and 5c), the presence of a tertiary amine and higher solvent polarity (ETN) play a more significant role in shifting the emission wavelength than the BTD core itself. This observation aligns with earlier studies,^58–60^ that state that tertiary amines enhance internal charge transfer (ICT), thereby creating a push–pull scenario that red shifts the emission spectra.^61^

For λ_max_^abs^, the absence of certain substructures clusters near zero SHAP values (Figure 4c), suggesting a negligible impact on the model’s predictions. Nevertheless, the presence of MF 1947, even if rare, significantly influences the outcome. This implies that a tertiary amine attached to both a benzene ring and a thiophene (forming a D-π-A system) can markedly boost λ_max_^abs^.

A high ETN value (indicating a more polar solvent) leads to an increase in the maximum emission wavelength (λ_max_^em^), causing a red-shift in the emission. This finding is consistent with Dyrager et al.,^62^ that elucidate the correlation between solvent polarity and Stokes shifts. Polar solvents stabilize the excited state more than the ground state, resulting in a red-shifted emission. Initially, the Franck–Condon excited state retains the ground-state solvation pattern,^32^ and as solvent molecules rearrange around the excited state’s dipole, they further stabilize it. The Lippert-Mataga equation quantifies this effect,^63–65^ showing that greater polarity enhances excited-state stabilization and shifts the emission toward the red region. The model successfully captures this behavior, demonstrating its capacity to incorporate and reflect fundamental chemical principles.

### Web Application

While computational chemistry and machine learning have significantly advanced the prediction of photophysical properties, these approaches often require specialized expertise in programming and data science, limiting accessibility for many researchers. To overcome this barrier and facilitate broader use of the developed predictive models, a user-friendly web application was built. This platform enables researchers without computational expertise to predict the photophysical properties of BTD derivatives easily. Additionally, the web application allows users to choose among the three machine learning models developed in this study—Random Forest, LightGBM, and XGBoost—as illustrated in Figure 6a. Users can input the molecular structure of their BTD derivative in two convenient ways: by entering the SMILES notation directly (Figure 6b) or using an integrated molecular drawing tool that generates the corresponding SMILES code (Figure 6c). This flexibility ensures that users can input structures even without prior knowledge of SMILES formatting.

**Figure 6:**
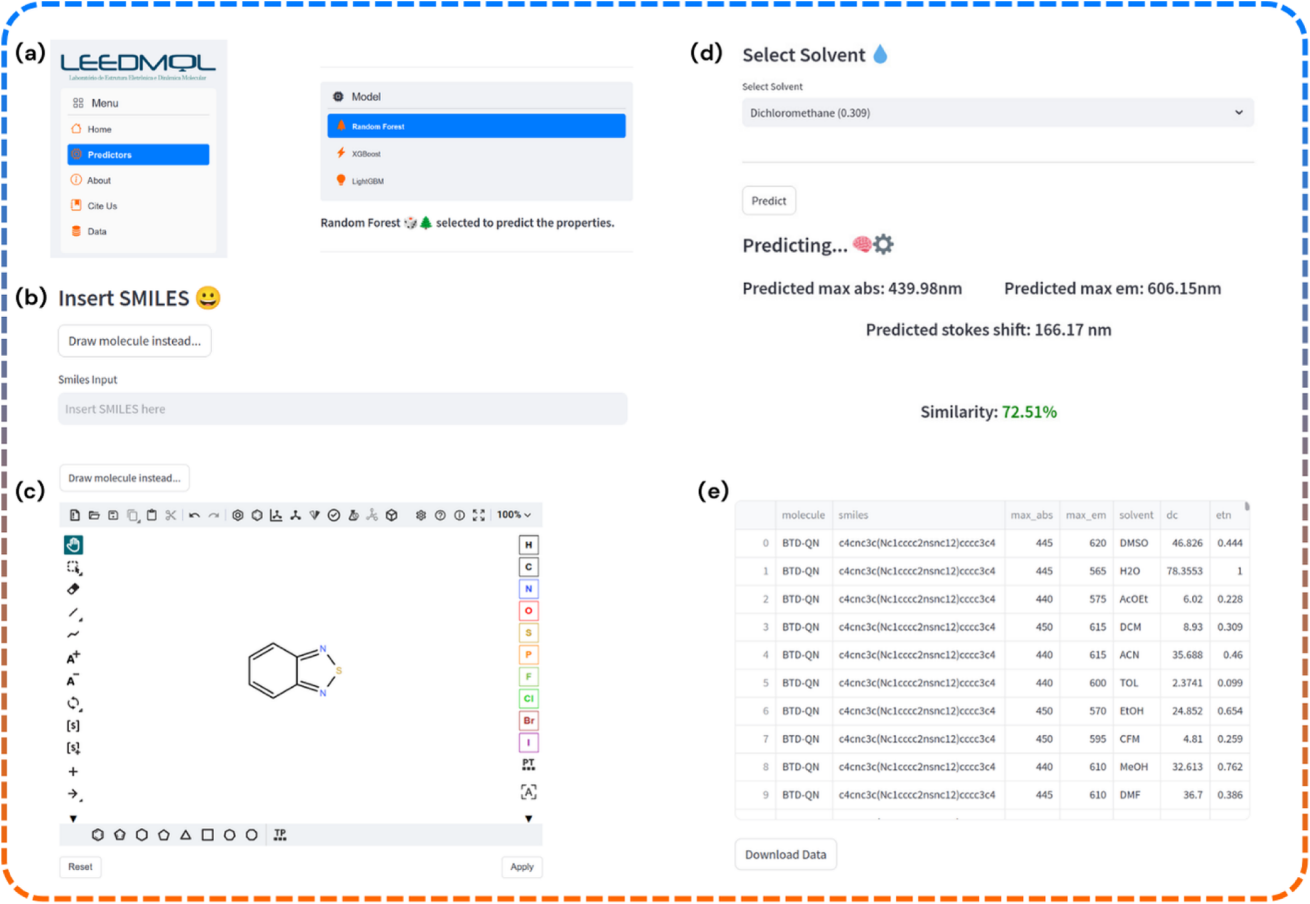
Overview of the WebApp workflow for predicting photophysical properties of benzothiadiazole derivatives. (a) Displays the main interface and the option to select predictive models. (b) Allows users to input SMILES or (c) draw molecular structures. (d) Presents the solvent selection and prediction results, including maximum absorption (λ_max_^abs^), emission (λ_max_^em^), Stokes shift, and structural similarity. (e) Presents the availability of the data set.

After specifying the molecular structure, users select a solvent from a list of predefined options present in the data set, accounting for solvent effects on photophysical properties. Upon submission, the application predicts the maximum absorption (λ_max_^abs^) and emission (λ_max_^em^) wavelengths using the selected model (Figure 6d). The application also provides information on the similarity between the user’s input molecule and compounds in the database, offering insights into the reliability of the predictions based on chemical space proximity. Furthermore, to support further analysis and integration with other tools, users can download the prediction results in CSV format (Figure 6e). The web application also ensures transparency by providing access to the underlying data set used to develop the models, enabling users to explore the data and understand the basis of the predictions. By making the predictive models accessible through this web application, a wider community of chemists, material scientists, and researchers in related fields can be empowered to explore and design new BTD derivatives with enhanced photophysical properties, without the need for extensive computational expertise. More recently, this tool not only democratizes access to advanced predictive modeling but also accelerates the discovery process by providing rapid, reliable predictions with minimal effort.

## Limitations

Our database is built from freely available literature sources, which impose limitations on its comprehensiveness and data balance. Notably, we observe an imbalance in the representation of solvents, with certain systems being more extensively studied and documented than others, potentially affecting the model’s applicability to less-explored contexts. Furthermore, our model is specifically focused on 2,1,3-benzothiadiazole (2,1,3-BTD), which may introduce limitations when applied to other BTD derivatives, as variations in their electronic and structural properties could lead to different behaviors not fully captured by the current data set. The web application is designed not to store any user data, ensuring privacy and security for all inputs, and the source code is freely available at https://github.com/LEEDMOL/BTD-JCIM-PAPER.

## Conclusions

This study demonstrates the effective integration of machine learning (ML) approaches and molecular fingerprints for predicting the photophysical properties of benzothiadiazole (BTD) derivatives, achieving high predictive accuracy and reduced computational costs. By employing algorithms such as Random Forest, LightGBM, and XGBoost, robust statistical metrics were obtained, including *R* ^2^, *Q* ^2^, MAE, and RMSE, that confirm the reliability and precision of the predictive models. Moreover, the use of SHapley Additive exPlanations (SHAP) provided clear mechanistic insights, revealing that tertiary amine substituents and solvent polarity play a decisive role in red-shifting emission wavelengths. These findings guide rational structural modifications and solvent choices, enabling more targeted and efficient photophysical optimization.

Looking ahead, the research will incorporate electron density-based descriptors derived from quantum-chemical calculations, complementing the conventional Morgan or MACCS fingerprints. This enhanced approach will offer a more nuanced atomic-level perspective on how substituents modulate the photophysical behavior. By merging ML-driven accuracy, detailed mechanistic interpretation through SHAP, and a deeper quantum-chemical understanding, future studies promise to accelerate the design of new BTD derivatives with finely tuned optical properties for cutting-edge photonic and optoelectronic applications.

## Supplementary Material



## Data Availability

The complete database used for training the models, along with the data cleaning scripts, model training code, and the trained models themselves, are all available at: https://github.com/LEEDMOL/BTD-JCIM-PAPER.

## Notes

Additionally, a web application has been developed to predict photophysical properties, which can be accessed via Streamlit cloud (PDF)

## References

[1] Miranda M. S., Matos M. A. R., Morais V. M., Liebman J. F. (2012). 2,1,3-Benzothiadiazole:
Study of its structure, energetics and aromaticity. J. Chem. Thermodyn..

[2] Neto B. A. D., Lapis A. A., da Silva Júnior E. N., Dupont J. (2013). 2, 1, 3-Benzothiadiazole
and derivatives: synthesis, properties, reactions, and applications
in light technology of small molecules. Eur.
J. Org. Chem..

[3] Sodre E. R., Guido B. C., de Souza P. E. N., Machado D. F. S., Carvalho-Silva V. H., Chaker J. A., Gatto C. C., Correa J. R., Fernandes T. d. A., Neto B. A. D. (2020). Deciphering the Dynamics of Organic Nanoaggregates
with AIEE Effect and Excited States: Lipophilic Benzothiadiazole Derivatives
as Selective Cell Imaging Probes. J. Org. Chem..

[4] Farré Y., Raissi M., Fihey A., Pellegrin Y., Blart E., Jacquemin D., Odobel F. (2018). Synthesis and properties
of new benzothiadiazole-based push-pull dyes for p-type dye sensitized
solar cells. Dyes Pigm..

[5] Wu J., Lai G., Li Z., Lu Y., Leng T., Shen Y., Wang C. (2016). Novel 2, 1, 3-benzothiadiazole
derivatives used as selective fluorescent
and colorimetric sensors for fluoride ion. Dyes
Pigm..

[6] Balasankar T., Gopalakrishnan M., Nagarajan S. (2005). Synthesis and antibacterial activity
of some 5-(4-biphenylyl)-7-aryl­[3,4-d] [1,2,3]-benzothiadiazoles. Eur. J. Med. Chem..

[7] DaSilveira
Neto B. A., Lopes A. S., Ebeling G., Gonçalves R. S., Costa V. E., Quina F. H., Dupont J. (2005). Photophysical and electrochemical
properties of *π*-extended molecular 2, 1, 3-benzothiadiazoles. Tetrahedron.

[8] Colas K., Holmberg K. O., Chiang L., Doloczki S., Swartling F. J., Dyrager C. (2021). Indolylbenzothiadiazoles
as highly tunable fluorophores
for imaging lipid droplet accumulation in astrocytes and glioblastoma
cells. RSC Adv..

[9] Ceriani C., Corsini F., Mattioli G., Mattiello S., Testa D., Po R., Botta C., Griffini G., Beverina L. (2021). Sustainable by design, large Stokes
shift benzothiadiazole
derivatives for efficient luminescent solar concentrators. J. Mater. Chem. C.

[10] Neto B. A. D., Carvalho P. H., Correa J. R. (2015). Benzothiadiazole derivatives as fluorescence
imaging probes: beyond classical scaffolds. Acc. Chem. Res..

[11] Dinçalp H., Murat G., İçli S. (2014). Improvement of intramolecular charge
transfer within a donor–acceptor blend by doping novel synthesized
benzothiadiazole small molecules in solid state. Opt. Mater..

[12] Bardi B., Dall’Agnese C., Ching K. I. M.-C., Painelli A., Terenziani F. (2017). Spectroscopic
investigation and theoretical modeling of benzothiadiazole-based charge-transfer
chromophores: From solution to nanoaggregates. J. Phys. Chem. C.

[13] Mohajeri A., Omidvar A., Setoodeh H. (2019). Fine Structural Tuning
of Thieno­[3,2-b]
Pyrrole Donor for Designing Banana-Shaped Semiconductors Relevant
to Organic Field Effect Transistors. J. Chem.
Inf. Model..

[14] Carvalho P. H. P. R., Correa J. R., Guido B. C., Gatto C. C., De Oliveira H. C., Soares T. A., Neto B. A. (2014). Designed benzothiadiazole fluorophores
for selective mitochondrial imaging and dynamics. Chem. – Eur. J..

[15] da
Cruz E. H. G., Carvalho P. H., Corrêa J. R., Silva D. A., Diogo E. B., de Souza Filho J. D., Cavalcanti B. C., Pessoa C., de Oliveira H. C., Guido B. C. (2014). Design, synthesis and application of fluorescent
2, 1, 3-benzothiadiazole-triazole-linked biologically active lapachone
derivatives. New J. Chem..

[16] Baranov M. S., Solntsev K. M., Baleeva N. S., Mishin A. S., Lukyanov S. A., Lukyanov K. A., Yampolsky I. V. (2014). Red-Shifted
Fluorescent Aminated
Derivatives of a Conformationally Locked GFP Chromophore. Chem. - Eur. J..

[17] Chen C., Fang C. (2020). Devising Efficient Red-Shifting Strategies for Bioimaging: A Generalizable
Donor-Acceptor Fluorophore Prototype. Chem.
- Asian J..

[18] Ceballos-Ávila D., Vázquez-Sandoval I., Ferrusca-Martınez F., Jiménez-Sánchez A. (2024). Conceptually innovative fluorophores
for functional bioimaging. Biosens. Bioelectron..

[19] Joung J. F., Han M., Hwang J., Jeong M., Choi D. H., Park S. (2021). Deep Learning
Optical Spectroscopy Based on Experimental Database: Potential Applications
to Molecular Design. JACS Au.

[20] Sanches-Neto F. O., Dias-Silva J. R., Junior L. H. K. Q., Carvalho-Silva V. H. (2021). “pySiRC”:
Machine Learning Combined with Molecular Fingerprints to Predict the
Reaction Rate Constant of the Radical-Based Oxidation Processes of
Aqueous Organic Contaminants. Environ. Sci.
Technol..

[21] Mahato K. D., Kumar U. (2024). Optimized machine learning techniques enable prediction of organic
dyes photophysical properties: Absorption wavelengths, emission wavelengths,
and quantum yields. Spectrochim. Acta, Part
A.

[22] Cereto-Massagué A., Ojeda M. J., Valls C., Mulero M., Garcia-Vallvé S., Pujadas G. (2015). Molecular fingerprint similarity search in virtual
screening. Methods.

[23] Zhong S., Hu J., Fan X., Yu X., Zhang H. (2020). A deep neural network
combined with molecular fingerprints (DNN-MF) to develop predictive
models for hydroxyl radical rate constants of water contaminants. J. Hazard. Mater..

[24] Ju C.-W., Bai H., Li B., Liu R. (2021). Machine learning
enables highly accurate
predictions of photophysical properties of organic fluorescent materials:
Emission wavelengths and quantum yields. J.
Chem. Inf. Model..

[25] Mai J., Lu T., Xu P., Lian Z., Li M., Lu W. (2022). Predicting
the maximum absorption wavelength of azo dyes using an interpretable
machine learning strategy. Dyes Pigm..

[26] Neto B. A. D., Carvalho P. H., Santos D. C., Gatto C. C., Ramos L. M., de Vasconcelos N. M., Corrêa J. R., Costa M. B., de Oliveira H. C., Silva R. G. (2012). Synthesis, properties and highly selective mitochondria
staining with novel, stable and superior benzothiadiazole fluorescent
probes. RSC Adv..

[27] Souza V. S., Corrêa J. R., Carvalho P. H., Zanotto G. M., Matiello G. I., Guido B. C., Gatto C. C., Ebeling G., Gonçalves P. F., Dupont J., Neto B. A. (2020). Appending ionic liquids to fluorescent
benzothiadiazole derivatives: Light up and selective lysosome staining. Sens. Actuators, B.

[28] Fonseca T., de Oliveira H., Castro M. (2008). Theoretical study of
the lowest electronic
transitions of sulfur-bearing mesoionic compounds in gas-phase and
in dimethyl sulfoxide. Chem. Phys. Lett..

[29] Neto A. P. V., Machado D. F. S., Lopes T. O., Camargo A. J., de Oliveira H. C. B. (2018). Explicit Aqueous Solvation Treatment
of Epinephrine
from Car–Parrinello Molecular Dynamics: Effect of Hydrogen
Bonding on the Electronic Absorption Spectrum. J. Phys. Chem. B.

[30] Lopes T. O., da Silva Filho D. A., Lapis A. A. M., de
Oliveira H. C. B., Neto B. A. D. (2014). Designed non-symmetrical 4,7-pi-extended-2,1,3-benzothiadiazole
derivatives: Synthesis guided by DFT predictions. J. Phys. Org. Chem..

[31] Dias-Silva J. R., Oliveira V. M., Sanches-Neto F. O., Wilhelms R. Z., Júnior L. H. Q. (2023). SpectraFP:
A new spectra-based descriptor to aid in cheminformatics, molecular
characterization and search algorithm applications. Phys. Chem. Chem. Phys..

[32] Reichardt C. (1994). Solvatochromic
Dyes as Solvent Polarity Indicators. Chem. Rev..

[33] Reichardt C., Harbusch-Görnert E. (1983). Über
Pyridinium-N-phenolat-Betaine
und ihre Verwendung zur Charakterisierung der Polarität von
Lösungsmitteln, X. Erweiterung, Korrektur und Neudefinition
der ET-Lösungsmittelpolaritätsskala mit Hilfe eines
lipophilen penta-tert-butyl-substituierten Pyridinium-N-phenolat-Betainfarbstoffes. Liebigs Ann. Chem..

[34] Weininger D. (1988). SMILES, a
chemical language and information system. 1. Introduction to methodology
and encoding rules. J. Chem. Inf. Comput. Sci..

[35] Landrum, G. rdkit/rdkit: 2024_09_2 (Q3 2024) Release. 2024.

[36] Morgan H. L. (1965). The Generation
of a Unique Machine Description for Chemical Structures-A Technique
Developed at Chemical Abstracts Service. J.
Chem. Doc..

[37] Martínez-Treviño S. H., Uc-Cetina V., Fernández-Herrera M. A., Merino G. (2020). Prediction
of Natural Product Classes Using Machine Learning and 13C NMR Spectroscopic
Data. J. Chem. Inf. Model..

[38] Mahato K. D., Das S. G. K., Azad C., Kumar U. (2024). Stokes shift prediction
of fluorescent organic dyes using machine learning based hybrid cascade
models. Dyes Pigm..

[39] Liao Z., Lu J., Xie K., Wang Y., Yuan Y. (2023). Prediction of Photochemical
Properties of Dissolved Organic Matter Using Machine Learning. Environ. Sci. Technol..

[40] Feitosa F. L., Cabral V. F., Sanches I. H., Silva-Mendonca S., Borba J. V. V. B., Braga R. C., Andrade C. H. (2024). Cyto-Safe: A Machine
Learning Tool for Early Identification of Cytotoxic Compounds in Drug
Discovery. J. Chem. Inf. Model..

[41] Mahato K. D., Das S. S. G. K., Azad C., Kumar U. (2024). Machine learning
based
hybrid ensemble models for prediction of organic dyes photophysical
properties: Absorption wavelengths, emission wavelengths, and quantum
yields. APL Mach. Learn..

[42] Chen, T. ; Guestrin, C. XGBoost: A Scalable Tree Boosting System, Proceedings of the 22nd ACM SIGKDD International Conference on Knowledge Discovery and Data Mining; ACM: New York, NY, USA, 2016; pp 785–794.

[43] Liaw A., Wiener M. (2002). Classification and
Regression by randomForest. R News.

[44] Ke, G. ; Meng, Q. ; Finley, T. ; Wang, T. ; Chen, W. ; Ma, W. ; Ye, Q. ; Liu, T.-Y. In LightGBM: A Highly Efficient Gradient Boosting Decision Tree, Advances in Neural Information Processing Systems 30 (NIPS 2017); NeurIPS, 2017.

[45] Welbl, J. Casting Random Forests as Artificial Neural Networks (and Profiting from It) Pattern Recognition.: Cham, 2014; pp 765–771.

[46] Li P., Zhang J.-S. (2018). A New Hybrid Method
for China’s Energy Supply
Security Forecasting Based on ARIMA and XGBoost. Energies.

[47] Bammou Y., Benzougagh B., Ouallali A., Kader S., Raougua M., Igmoullan B. (2025). Improving landslide susceptibility mapping in semi-arid
regions using machine learning and geospatial techniques. DYSONA - Appl. Sci..

[48] do
Casal M. T., Veys K., Bousquet M. H. E., Escudero D., Jacquemin D. (2023). First-Principles Calculations of Excited-State Decay
Rate Constants in Organic Fluorophores. J. Phys.
Chem. A.

[49] Pedregosa F. (2011). Scikit-learn: Machine Learning in Python. J.
Mach. Learn. Research.

[50] Akiba, T. ; Sano, S. ; Yanase, T. ; Ohta, T. ; Koyama, M. In Optuna: A Next-generation Hyperparameter Optimization Framework, Proceedings of the 25th ACM SIGKDD International Conference on Knowledge Discovery and Data Mining; ACM, 2019.

[51] Strieth-Kalthoff F., Sandfort F., Segler M. H. S., Glorius F. (2020). Machine learning the
ropes: principles, applications and directions in synthetic chemistry. Chem. Soc. Rev..

[52] Rücker C., Rücker G., Meringer M. (2007). y-Randomization and Its Variants
in QSPR/QSAR. J. Chem. Inf. Model..

[53] Kiralj R., Ferreira M. M. C. (2009). Basic validation
procedures for regression models in
QSAR and QSPR studies: theory and application. J. Braz. Chem. Soc..

[54] OECD . Guidance Document on the Validation of (Quantitative) Structure-Activity Relationship [(Q)­SAR] Models. 2014, p 154.

[55] Togo M. V., Mastrolorito F., Ciriaco F., Trisciuzzi D., Tondo A. R., Gambacorta N., Bellantuono L., Monaco A., Leonetti F., Bellotti R., Altomare C. D., Amoroso N., Nicolotti O. (2023). TIRESIA: An eXplainable Artificial
Intelligence Platform for Predicting Developmental Toxicity. J. Chem. Inf. Model..

[56] Lundberg S. M., Erion G., Chen H., DeGrave A., Prutkin J. M., Nair B., Katz R., Himmelfarb J., Bansal N., Lee S.-I. (2020). From local explanations to global
understanding with explainable AI for trees. Nat. Mach. Intell..

[57] Lundberg, S. M. ; Erion, G. G. ; Lee, S.-I. Consistent Individualized Feature Attribution for Tree Ensembles, 2019. arXiv:1802.03888. arXiv.org e-Print archive. https://arxiv.org/abs/1802.03888.

[58] Appelqvist H., Stranius K., Börjesson K., Nilsson K. P. R., Dyrager C. (2017). Specific Imaging
of Intracellular Lipid Droplets Using a Benzothiadiazole Derivative
with Solvatochromic Properties. Bioconjugate
Chem..

[59] Colas K., Doloczki S., Kesidou A., Sainero-Alcolado L., Rodriguez-Garcia A., Arsenian-Henriksson M., Dyrager C. (2021). Photophysical Characteristics
of Polarity-Sensitive and Lipid Droplet-Specific Phenylbenzothiadiazoles. ChemPhotoChem.

[60] Dyrager C., Vieira R. P., Nyström S., Nilsson K. P. R., Storr T. (2017). Synthesis
and evaluation of benzothiazole-triazole and benzothiadiazole-triazole
scaffolds as potential molecular probes for amyloid-Beta aggregation. New J. Chem..

[61] Thomas K. R. J., Lin J. T., Velusamy M., Tao Y.-T., Chuen C.-H. (2004). Color Tuning
in Benzo­[1,2,5]­thiadiazole-Based Small Molecules by Amino Conjugation/Deconjugation:
Bright Red-Light-Emitting Diodes. Adv. Funct.
Mater..

[62] Doloczki S., Holmberg K. O., Galván I. F., Swartling F. J., Dyrager C. (2022). Photophysical characterization and fluorescence cell
imaging applications of 4-N-substituted benzothiadiazoles. RSC Adv..

[63] Lippert E. (1957). Spektroskopische
Bestimmung des Dipolmomentes aromatischer Verbindungen im ersten angeregten
Singulettzustand. Z. Elektrochem., Ber. Bunsengesellschaft
Phys. Chem..

[64] Mataga N., Kaifu Y., Koizumi M. (1956). Solvent Effects upon Fluorescence
Spectra and the Dipolemoments of Excited Molecules. Bull. Chem. Soc. Jpn..

[65] Krebs F. C., Spanggaard H. (2002). An Exceptional Red Shift of Emission Maxima upon Fluorine
Substitution. J. Org. Chem..

