# Supplementary material for: Integrating Machine Learning and SHAP Analysis to Advance the Rational Design of Benzothiadiazole Derivatives with Tailored Photophysical Properties
Source: J Chem Inf Model. 2025 Apr 29;65(15):7874–86. doi: 10.1021/acs.jcim.4c02414 (PMC12344762; doi:10.1021/acs.jcim.4c02414)
Supplement: Supplementary file 1 [file ci4c02414_si_001.pdf]

# Integrating Machine Learning and SHAP Analysis to Advance the Rational Design of Benzothiadiazole Derivatives with Tailored Photophysical Properties

Rafael F. Veríssimo,[†] Pedro H. F. Matias,[†] Mateus R. Barbosa,[†] Flávio O. S. Neto,[†,‡] Brenno A. D. Neto,[¶] and Heibbe C. B. de Oliveira[*,†]

†*Laboratório de Estrutura Eletrônica e Dinâmica Molecular, Universidade Federal de Goiás, Goiânia, GO, Brasil.*

‡*Instituto Federal de Educação, Ciência e Tecnologia de Goiás, 72876-601, Valparaíso de Goiás, GO, Brasil.*

¶*Laboratório de Química Medicinal e Tecnológica, Universidade De Brasília, Institute of Chemistry, 70910-900, Brasília, DF, Brasil.*

E-mail: heibbe@ufg.br

# Contents


**Models** **S4**

Random Forest . . . . . . . . . . . . . . . . . . . . . . . . . . . . . . . . . . . S4

eXtreme Gradient Boosting . . . . . . . . . . . . . . . . . . . . . . . . . . . . S4

Light Gradient Boosting Machine . . . . . . . . . . . . . . . . . . . . . . . . . S4

**Fingerprint Length and Radius** **S5**

Figure S1: Correlation plot between the fingerprint bit, its radius, and the $R^2$ coefficient from model training using XGBoost. . . . . . . . . . . . . . . . . . S5

**Hyperparameters** **S5**

Table S1: Fine-tuned hyperparameters obtained through Optuna optimization for eXtreme Gradient Boosting (XGBoost). . . . . . . . . . . . . . . . . . . . . S5

**Y randomization** **S6**

Figure S2: $R^2$ and $Q^2$ scores across 10 iterations for the y-randomization test for the Random Forest model predicting $\lambda_{max}^{abs}$, demonstrating significantly worse performance metrics compared to the original model, thus validating its robustness and generalization capability. . . . . . . . . . . . . . . . . . . S7

Figure S3: $R^2$ and $Q^2$ scores across 10 iterations for the y-randomization test for the Random Forest model predicting $\lambda_{max}^{em}$, demonstrating significantly worse performance metrics compared to the original model, thus validating its robustness and generalization capability. . . . . . . . . . . . . . . . . . . S8

**Scatter Plots** **S8**

Figure S4: Predicted values versus experimental values for a) max absorption and b) max emission for LGBM, red line represents the ideal prediction and blue points the actual prediction. . . . . . . . . . . . . . . . . . . . . . . . . . . S9

Figure S5: Predicted values versus experimental values for a) max absorption and b) max emission for XGBoost, red line represents the ideal prediction and blue points the actual prediction. . . . . . . . . . . . . . . . . . . . . . . . . S10

**Residuals** **S10**

Figure S6: Residual plot showing the difference between predicted and expected values for the maximum absorption wavelengths (nm). The model captures the overall trends, with deviations primarily concentrated around the expected values. . . . . . . . . . . . . . . . . . . . . . . . . . . . . . . . . . . . . . . S11

Figure S7: Residual plot showing the difference between predicted and expected values for the maximum emission wavelengths (nm). The model demonstrates good generalization, with residuals scattered closely around the expected values.S11

**External Testing** **S11**

Table S2: External testing of the model in predicting max absorption and max emission with experimental data from the literature. Experimental values (Exp) were measured in different solvents: [A] Tetrahydrofuran (THF), [B] Toluene (TOL), [C] Methanol (MeOH), and [D] Dichloromethane (DCM). Predicted values (Pred) represent the model's outputs, for LGBM . . . . . . S14

Table S3: External testing of the model in predicting max absorption and max emission with experimental data from the literature. Experimental values (Exp) were measured in different solvents: [A] Tetrahydrofuran (THF), [B] Toluene (TOL), [C] Methanol (MeOH), and [D] Dichloromethane (DCM). Predicted values (Pred) represent the model's outputs, for XGBoost . . . . . S16

**SHAP** **S17**

Figure S8: Visualization of SHAP analysis and molecular features influencing the predictive $\lambda_{max}^{abs}$ model. (a) Mean SHAP values of top molecular features (MFs) highlight their overall importance. (b) Bee swarm plot showing SHAP values for each molecular feature with color indicating feature magnitude (high: red, low: blue). (c) Molecular structures of selected features, classified as positively (+) or negatively (-) contributing to the model's output. For XGBoost. . . . S18

Figure S9: Visualization of SHAP analysis and molecular features influencing the predictive $\lambda_{max}^{em}$ model. (a) Mean SHAP values of top molecular features (MFs) highlight their overall importance. (b) Bee swarm plot showing SHAP values for each molecular feature with color indicating feature magnitude (high: red, low: blue). (c) Molecular structures of selected features, classified as positively (+) or negatively (-) contributing to the model's output. For XGBoost. . . . S19

Figure S10: Visualization of SHAP analysis and molecular features influencing the predictive $\lambda_{max}^{abs}$ model. (a) Mean SHAP values of top molecular features (MFs) highlight their overall importance. (b) Bee swarm plot showing SHAP values for each molecular feature with color indicating feature magnitude (high: red, low: blue). (c) Molecular structures of selected features, classified as positively (+) or negatively (-) contributing to the model's output. For LGBM. . . . . S20

Figure S11: Visualization of SHAP analysis and molecular features influencing the predictive $\lambda_{max}^{em}$ model. (a) Mean SHAP values of top molecular features (MFs) highlight their overall importance. (b) Bee swarm plot showing SHAP values for each molecular feature with color indicating feature magnitude (high: red, low: blue). (c) Molecular structures of selected features, classified as positively (+) or negatively (-) contributing to the model's output. For LGBM. . . . . S20

# Models

## Random Forest

Random Forest (RF) is a widely used algorithm for regression and classification models.[1,2] It is an ensemble method composed of multiple decision trees, each built using a subset of randomly selected variables. The incorporation of randomness in the tree construction enhances model diversity, leading to improved performance compared to other decision tree-based models.[1,3,4]

## eXtreme Gradient Boosting

Another algorithm used is eXtreme Gradient Boosting (XGBoost), which is based on gradient-boosting decision trees.[5] Developed within the Gradient Boosting framework, XGBoost is designed for high efficiency, flexibility, and portability. Its parallel tree reinforcement capability enables rapid and accurate solutions to various data science problems, making it a widely used approach in recent studies.[6–9]

## Light Gradient Boosting Machine

An additional gradient boost framework that offers high efficiency and scalability is the Light Gradient Boosting Machine (LGBM). Unlike traditional gradient-boosting algorithms, LGBM utilizes a histogram-based learning approach, which significantly reduces memory usage and computational time. This method enables faster training while maintaining high predictive accuracy.[10]

LGBM constructs decision trees in a leaf-wise manner rather than a level-wise approach, allowing deeper, more optimal splits that enhance model performance.[11] In addition, it incorporates techniques such as gradient-based one-side sampling (GOSS) and exclusive feature bundle (EFB), which further improve efficiency by reducing the number of data points and features processed during training.[10]

# Fingerprint Length and Radius

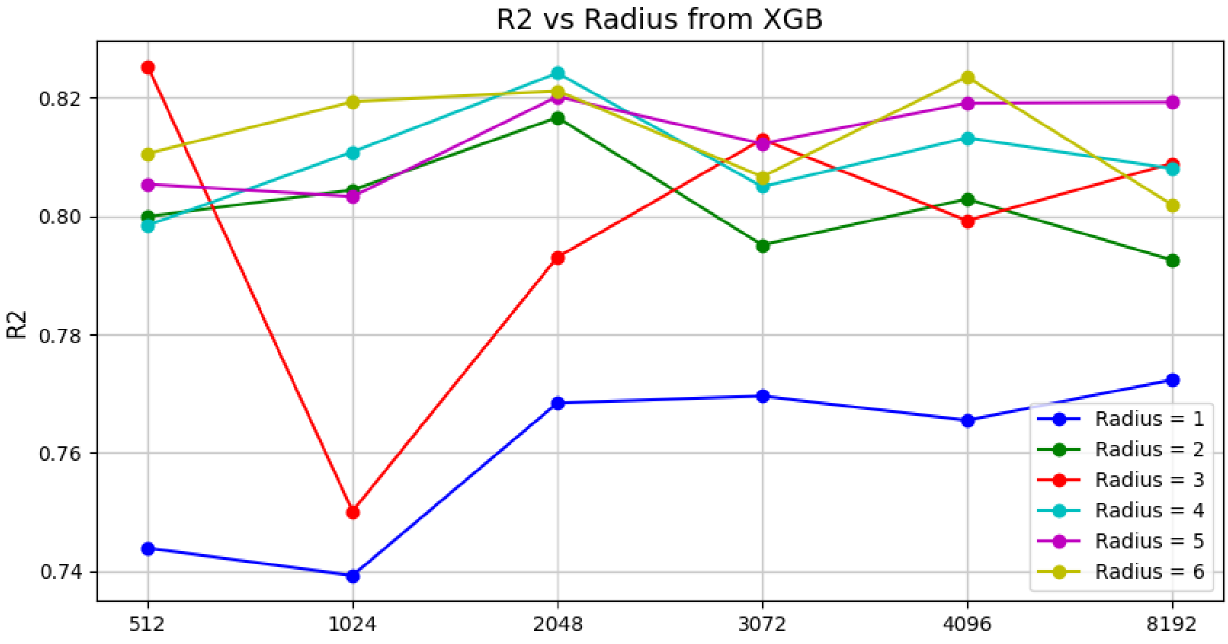


Figure S1: Correlation plot between the fingerprint bit, its radius, and the $R^2$ coefficient from model training using XGBoost.

# Hyperparameters

For random forest (RF) and light gradient booster machine (LGBM), Optuna did not produce acceptable results. Therefore, the default parameters were used for RF and LGBM.

Table S1: Fine-tuned hyperparameters obtained through Optuna optimization for eXtreme Gradient Boosting (XGBoost).

| **Hyperparameter** | $\lambda_{\mathbf{max}}^{\mathbf{abs}}$ | $\lambda_{\mathbf{max}}^{\mathbf{em}}$ |
|---|---|---|
| n_estimators | 522 | 502 |
| max_depth | 8 | 7 |
| learning_rate | 0.0286 | 0.0331 |
| subsample | 0.8384 | 0.7929 |
| colsample_bytree | 0.7201 | 0.7203 |
| gamma | 0.0018 | $2.7 \times 10^{-6}$ |
| reg_alpha | $7.71 \times 10^{-5}$ | $2.15 \times 10^{-6}$ |
| reg_lambda | 0.0995 | $1.08 \times 10^{-4}$ |
| min_child_weight | 4 | 5 |

# Y randomization

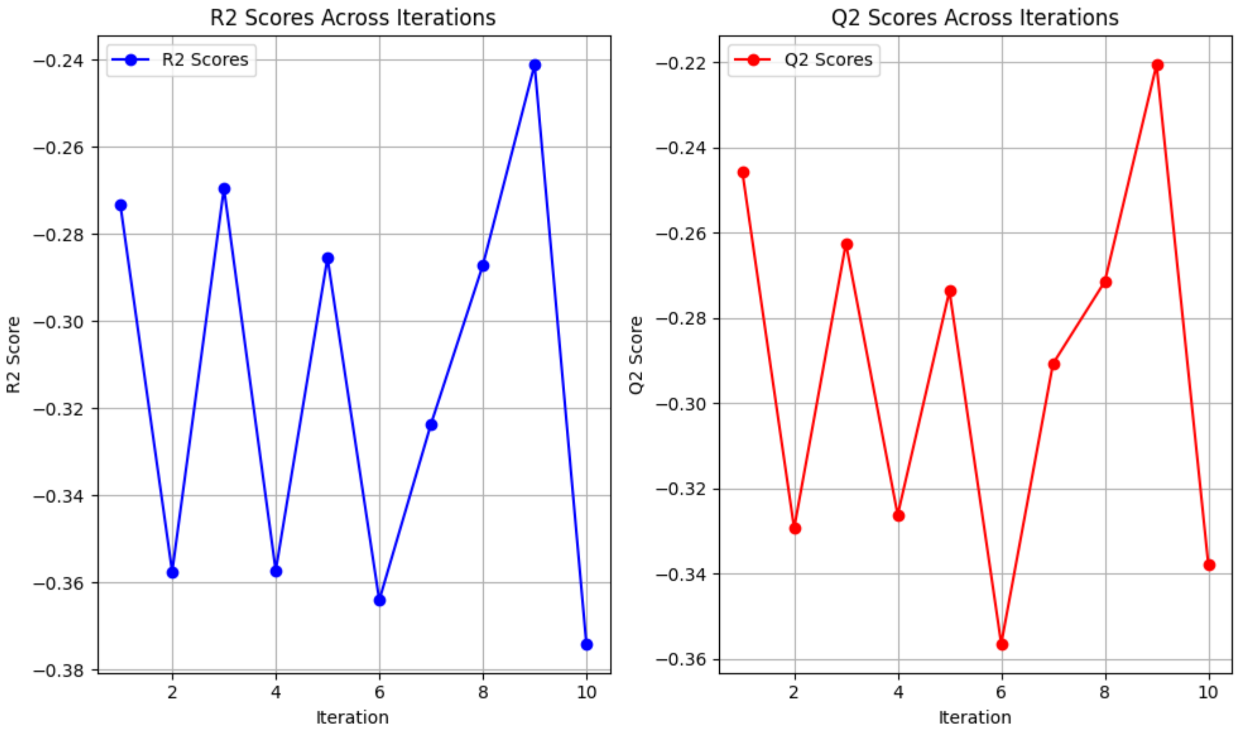


Figure S2: $R^2$ and $Q^2$ scores across 10 iterations for the y-randomization test for the Random Forest model predicting $\lambda_{max}^{abs}$, demonstrating significantly worse performance metrics compared to the original model, thus validating its robustness and generalization capability.

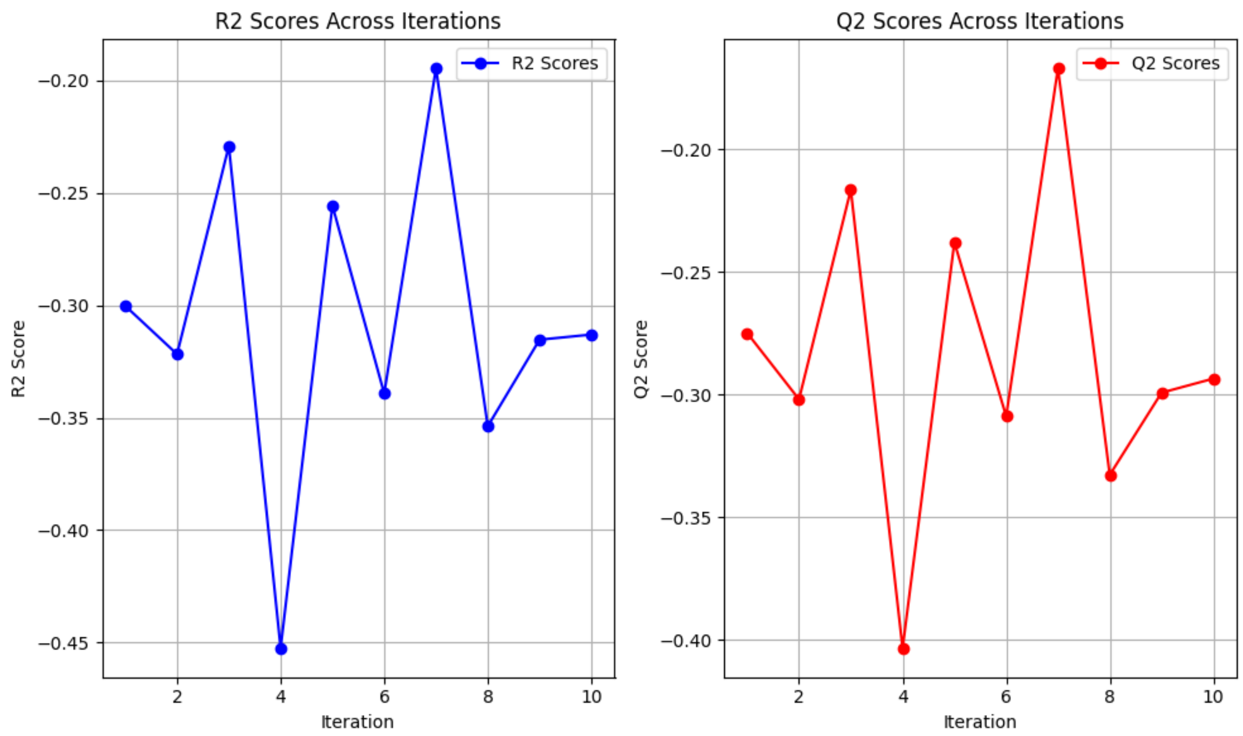


Figure S3: $R^2$ and $Q^2$ scores across 10 iterations for the y-randomization test for the Random Forest model predicting $\lambda_{max}^{em}$, demonstrating significantly worse performance metrics compared to the original model, thus validating its robustness and generalization capability.

# Scatter Plots

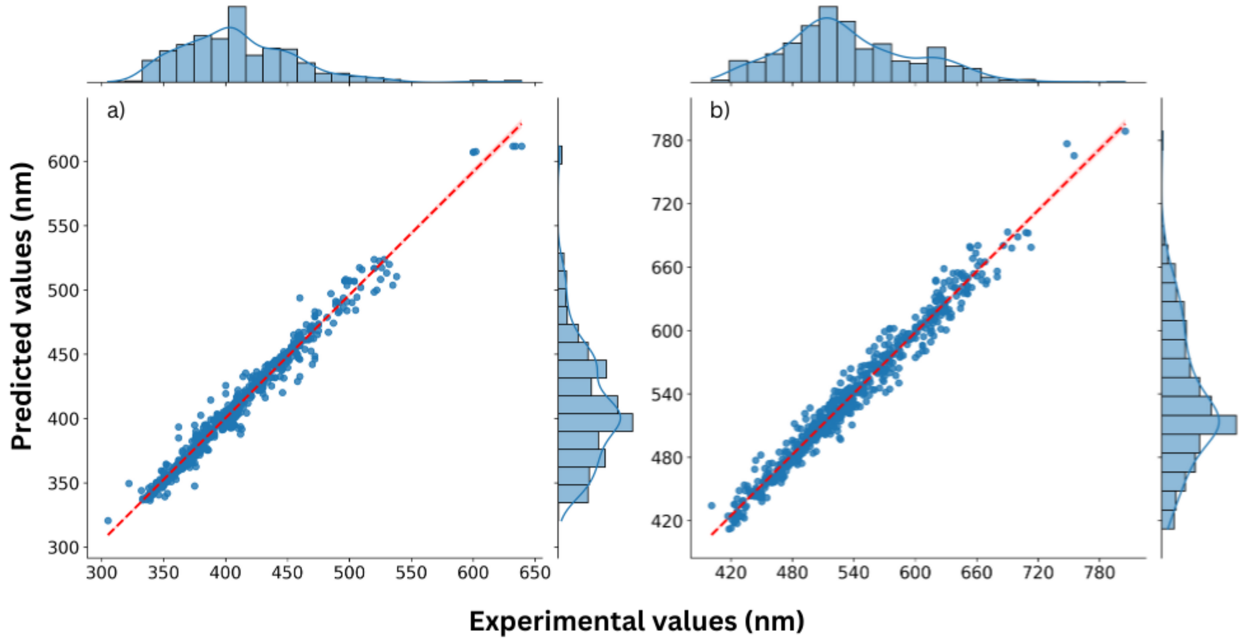


Figure S4: Predicted values versus experimental values for a) max absorption and b) max emission for LGBM, red line represents the ideal prediction and blue points the actual prediction.

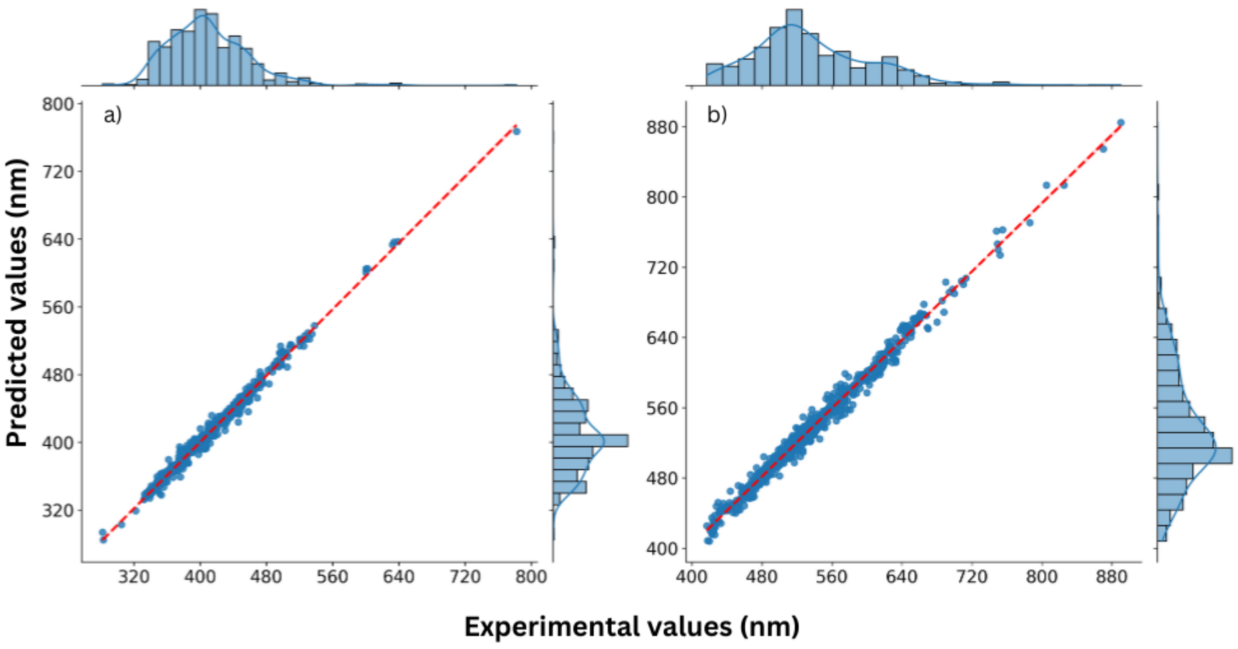


Figure S5: Predicted values versus experimental values for a) max absorption and b) max emission for XGBoost, red line represents the ideal prediction and blue points the actual prediction.

# Residuals

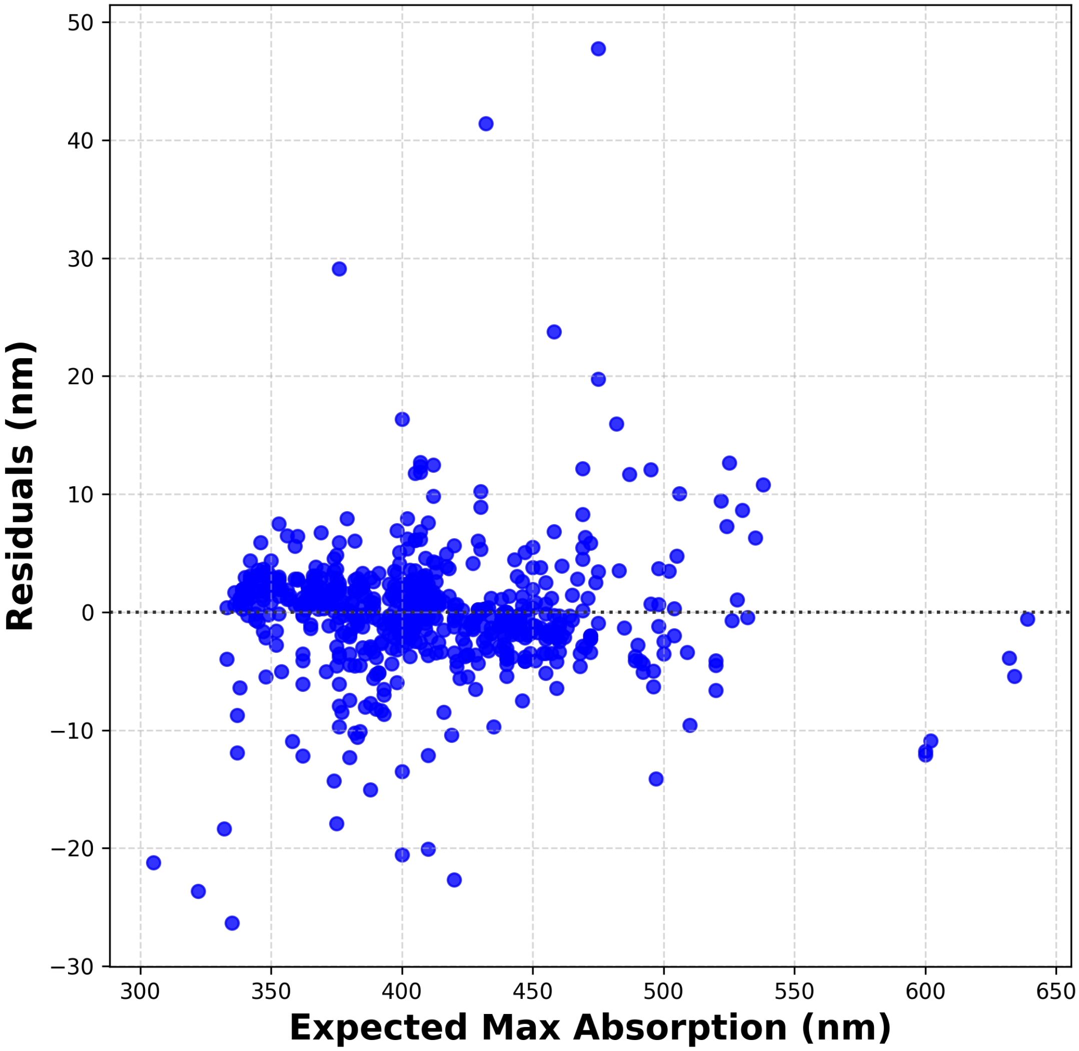


Figure S6: Residual plot showing the difference between predicted and expected values for the maximum absorption wavelengths (nm). The model captures the overall trends, with deviations primarily concentrated around the expected values.

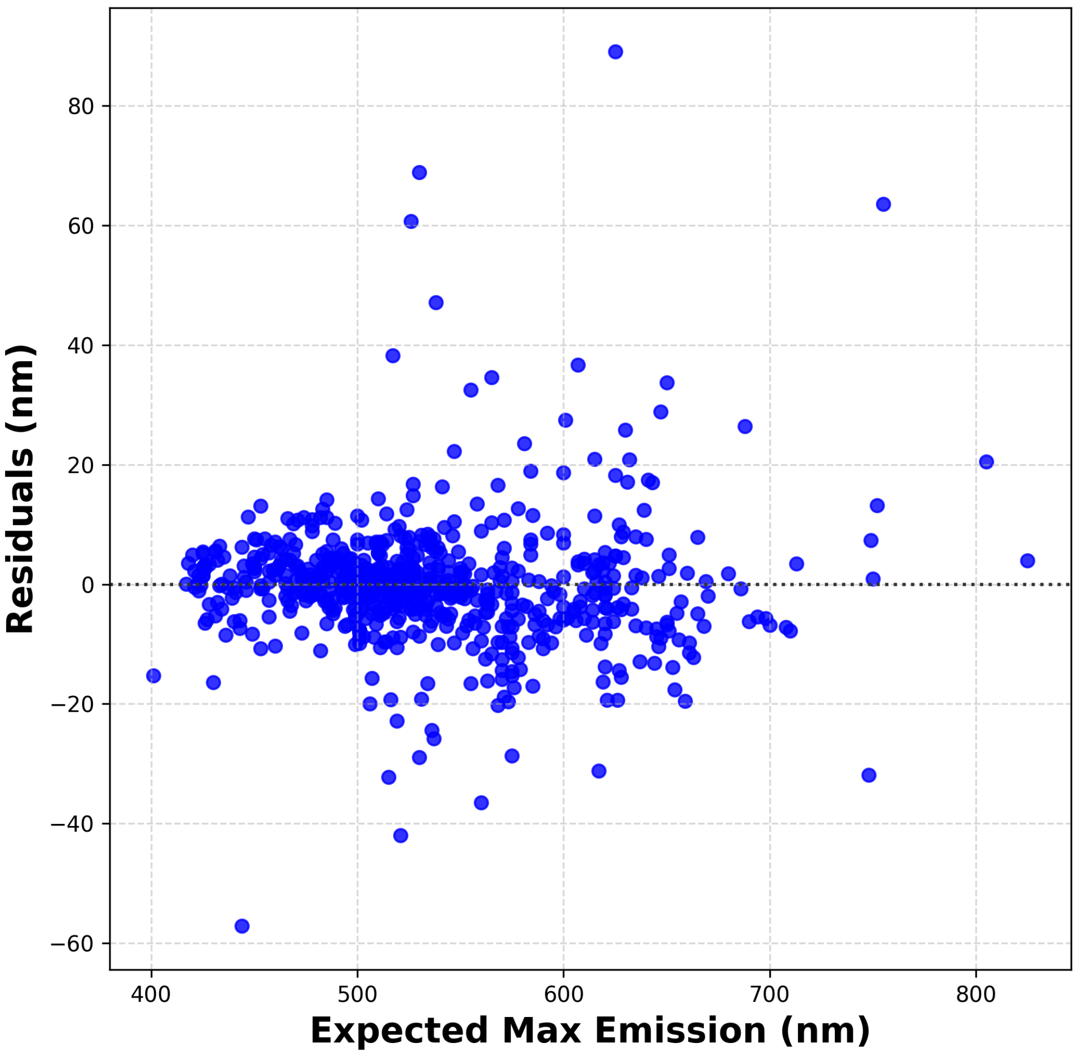


Figure S7: Residual plot showing the difference between predicted and expected values for the maximum emission wavelengths (nm). The model demonstrates good generalization, with residuals scattered closely around the expected values.

# External Testing

Table S2: External testing of the model in predicting max absorption and max emission with experimental data from the literature. Experimental values (Exp) were measured in different solvents: [A] Tetrahydrofuran (THF), [B] Toluene (TOL), [C] Methanol (MeOH), and [D] Dichloromethane (DCM). Predicted values (Pred) represent the model's outputs, for LGBM

| Molecules | Max Absorption | | Max Emission | | Similarity |
|---|---|---|---|---|---|
| | Exp(nm) | Pred(nm)/Error | Exp(nm) | Pred(nm)/Error | |
| 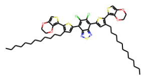 | 399.00 | 351.34 / 11.94% | 514.00 | 468.42 / 8.87% | 99.73 |
| 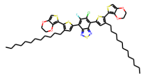 | 364.00 | 369.54 / 1.52% | 475.00 | 472.40 / 0.55% | 97.46 |
| 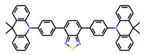 | 323.00 | 363.65 / 12.59% | 432.00 | 506.01 / 17.13% | 74.43 |
| 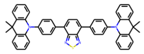 | 325.00 | 367.91 / 13.20% | 400.00 | 478.93 / 19.73% | 74.43 |
| 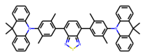 | 490.00 | 460.43 / 6.03% | 634.00 | 598.38 / 5.62% | 72.51 |
| 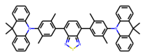 | 471.00 | 449.58 / 4.55% | 632.00 | 586.77 / 7.16% | 69.80 |
| 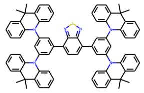 | 381.00 | 379.61 / 0.36% | 738.00 | 528.98 / 28.32% | 66.90 |
| 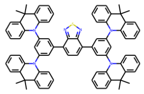 | 382.00 | 381.41 / 0.15% | 574.00 | 521.10 / 9.22% | 66.90 |

Continued on next page

Continuation of Table S2

| Molecules | Max Absorption | | Max Emission | | Similarity |
|---|---|---|---|---|---|
| | Exp(nm) | Pred(nm)/Error | Exp(nm) | Pred(nm)/Error | |
| 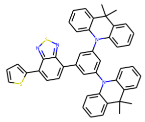 | 418.00 | 393.33 / 5.90% | 610.00 | 549.48 / 9.92% | 63.85 |
| 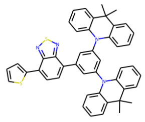 | 416.00 | 393.45 / 5.42% | 766.00 | 561.48/ 26.70% | 63.85 |
| 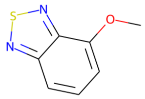 | 356.00 | 398.37 / 11.90% | 540.00 | 547.04 / 1.30% | 63.80 |
| 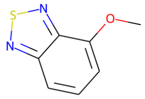 | 357.00 | 400.97 / 12.32% | 728.00 | 550.05 / 24.44% | 63.80 |
| 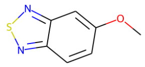 | 353.00 | 404.48 / 14.58% | 484.00 | 523.17 / 8.09% | 63.55 |
| 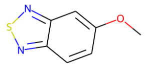 | 353.00 | 398.22 / 12.81% | 533.00 | 588.68 / 10.45% | 63.55 |
| 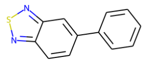 | 379.00 | 381.59 / 0.68% | 622.00 | 542.09 / 12.85% | 63.19 |
| 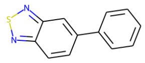 | 377.00 | 381.84 / 1.28% | 765.00 | 545.40 / 28.71% | 63.19 |
| 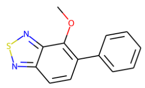 | 338.00 | 405.49 / 19.97% | 391.00 | 516.32 / 32.05% | 56.94 |
| 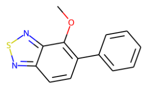 | 337.00 | 407.01 / 20.77% | 421.00 | 545.58 / 29.59% | 56.94 |

Continued on next page

Continuation of Table S2

| Molecules | Max Absorption | | Max Emission | | Similarity |
|---|---|---|---|---|---|
| | Exp(nm) | Pred(nm)/Error | Exp(nm) | Pred(nm)/Error | |
| 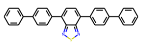 | 360.00 | 400.27 / 11.19% | 501.00 | 573.38 / 14.45% | 50.17 |
| 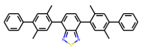 | 357.00 | 402.55 / 12.76% | 445.00 | 527.18 / 18.47% | 50.17 |

Table S3: External testing of the model in predicting max absorption and max emission with experimental data from the literature. Experimental values (Exp) were measured in different solvents: [A] Tetrahydrofuran (THF), [B] Toluene (TOL), [C] Methanol (MeOH), and [D] Dichloromethane (DCM). Predicted values (Pred) represent the model's outputs, for XGBoost

| Molecules | Max Absorption | | Max Emission | | Similarity |
|---|---|---|---|---|---|
| | Exp(nm) | Pred(nm)/Error | Exp(nm) | Pred(nm)/Error | |
| 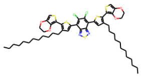 | 399.00 | 394.05 / 1.24% | 514.00 | 489.89 / 4.69% | 99.73 |
| 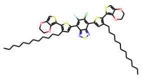 | 364.00 | 400.52 / 10.03% | 475.00 | 508.84 / 7.13% | 97.46 |
| 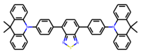 | 323.00 | 397.27 / 22.99% | 432.00 | 498.10 / 15.30% | 74.43 |
| 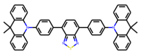 | 325.00 | 396.13 / 21.89% | 400.00 | 466.03 / 16.51% | 74.43 |
| 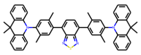 | 490.00 | 472.53 / 3.56% | 634.00 | 604.38 / 4.67% | 72.51 |

Continued on next page

Continuation of Table S3

| Molecules | Max Absorption | | Max Emission | | Similarity |
|---|---|---|---|---|---|
| | Exp(nm) | Pred(nm)/Error | Exp(nm) | Pred(nm)/Error | |
| 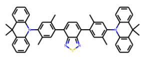 | 471.00 | 516.61 / 9.68% | 632.00 | 626.10 / 0.93% | 69.80 |
| 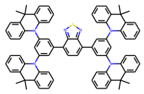 | 381.00 | 396.10 / 3.96% | 738.00 | 573.63 / 22.27% | 66.90 |
| 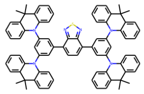 | 382.00 | 395.23 / 3.46% | 574.00 | 558.06 / 2.78% | 66.90 |
| 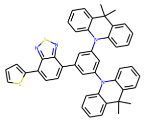 | 418.00 | 412.93 / 1.21% | 610.00 | 578.71 / 5.13% | 63.85 |
| 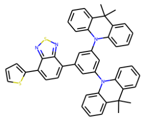 | 416.00 | 411.09 / 1.18% | 766.00 | 601.74 / 21.44% | 63.85 |
| 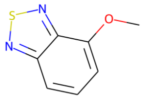 | 356.00 | 407.95 / 14.59% | 540.00 | 560.06 / 3.72% | 63.80 |
| 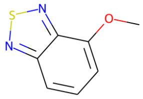 | 357.00 | 409.38 / 14.67% | 728.00 | 574.30 / 21.11% | 63.80 |
| 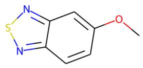 | 353.00 | 413.55 / 17.15% | 484.00 | 545.15 / 12.64% | 63.55 |
| 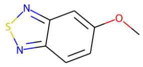 | 353.00 | 417.18 / 18.18% | 533.00 | 602.97 / 13.13% | 63.55 |
| 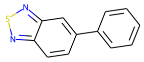 | 379.00 | 401.17 / 5.85% | 622.00 | 568.20 / 8.65% | 63.19 |

Continued on next page

Continuation of Table S3

| Molecules | Max Absorption | | Max Emission | | Similarity |
|---|---|---|---|---|---|
| | Exp(nm) | Pred(nm)/Error | Exp(nm) | Pred(nm)/Error | |
| 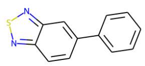 | 377.00 | 400.98 / 6.36% | 765.00 | 583.44 / 23.73% | 63.19 |
| 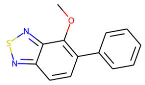 | 338.00 | 401.46 / 18.77% | 391.00 | 504.72 / 29.09% | 56.94 |
| 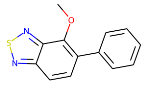 | 337.00 | 402.54 / 19.45% | 421.00 | 533.88 / 26.81% | 56.94 |
| 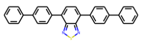 | 360.00 | 405.87 / 12.74% | 501.00 | 549.95 / 9.77% | 50.17 |
| 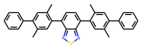 | 357.00 | 406.39 / 13.84% | 445.00 | 519.00 / 16.63% | 50.17 |

# SHAP

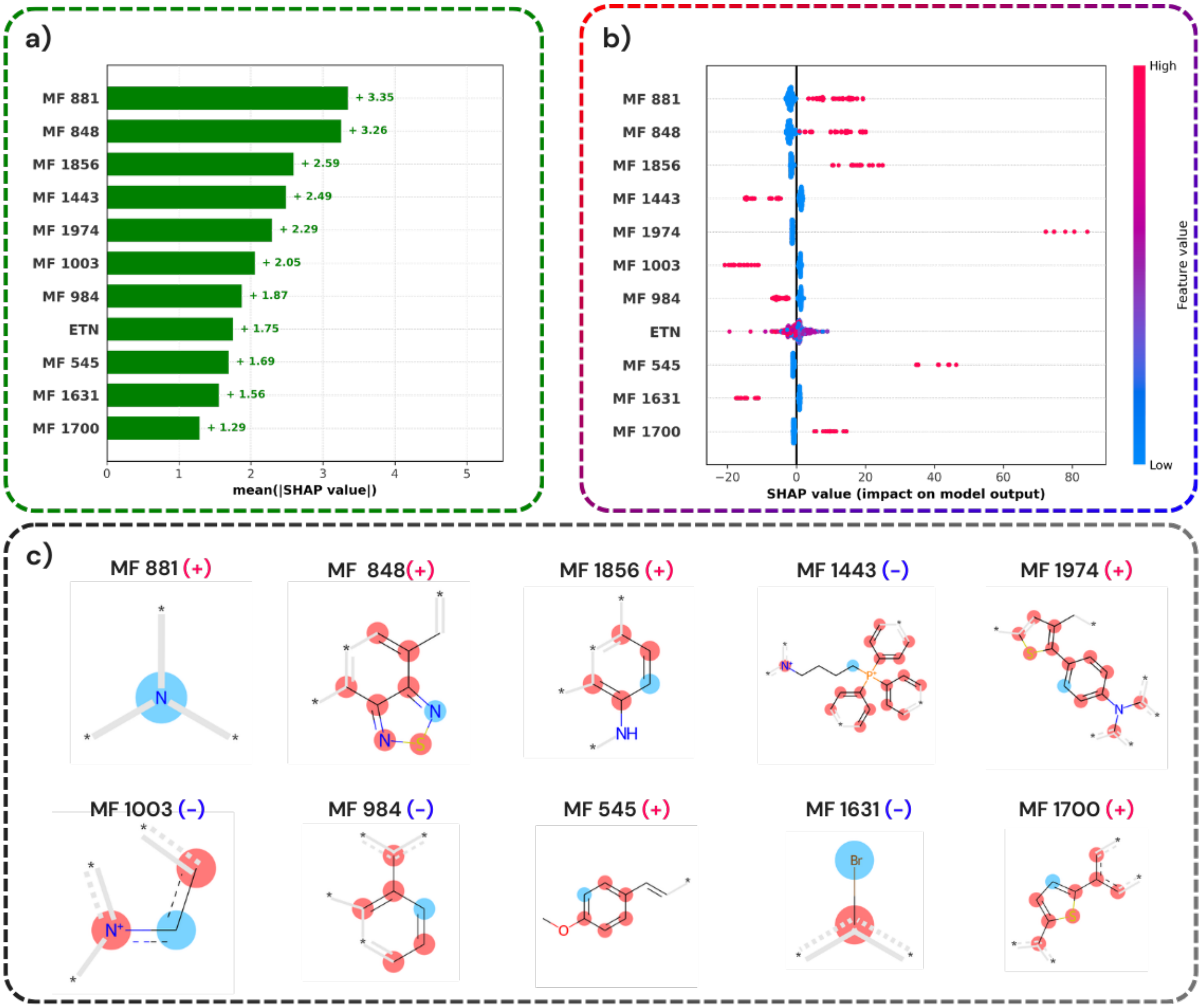


Figure S8: Visualization of SHAP analysis and molecular features influencing the predictive $\lambda_{max}^{abs}$ model. (a) Mean SHAP values of top molecular features (MFs) highlight their overall importance. (b) Bee swarm plot showing SHAP values for each molecular feature with color indicating feature magnitude (high: red, low: blue). (c) Molecular structures of selected features, classified as positively (+) or negatively (-) contributing to the model's output. For XGBoost

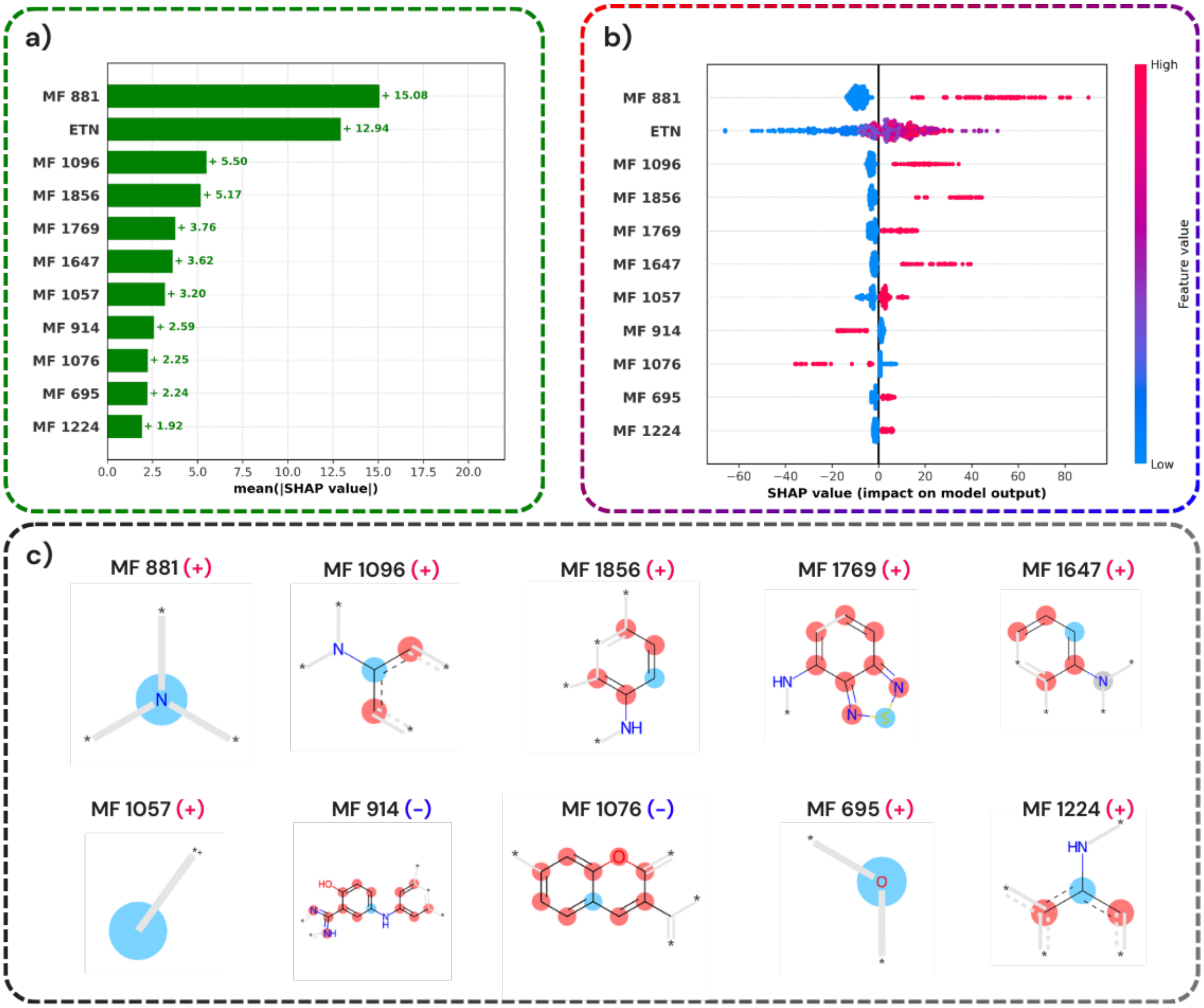


Figure S9: Visualization of SHAP analysis and molecular features influencing the predictive $\lambda_{max}^{em}$ model. (a) Mean SHAP values of top molecular features (MFs) highlight their overall importance. (b) Bee swarm plot showing SHAP values for each molecular feature with color indicating feature magnitude (high: red, low: blue). (c) Molecular structures of selected features, classified as positively (+) or negatively (-) contributing to the model's output. For XGBoost

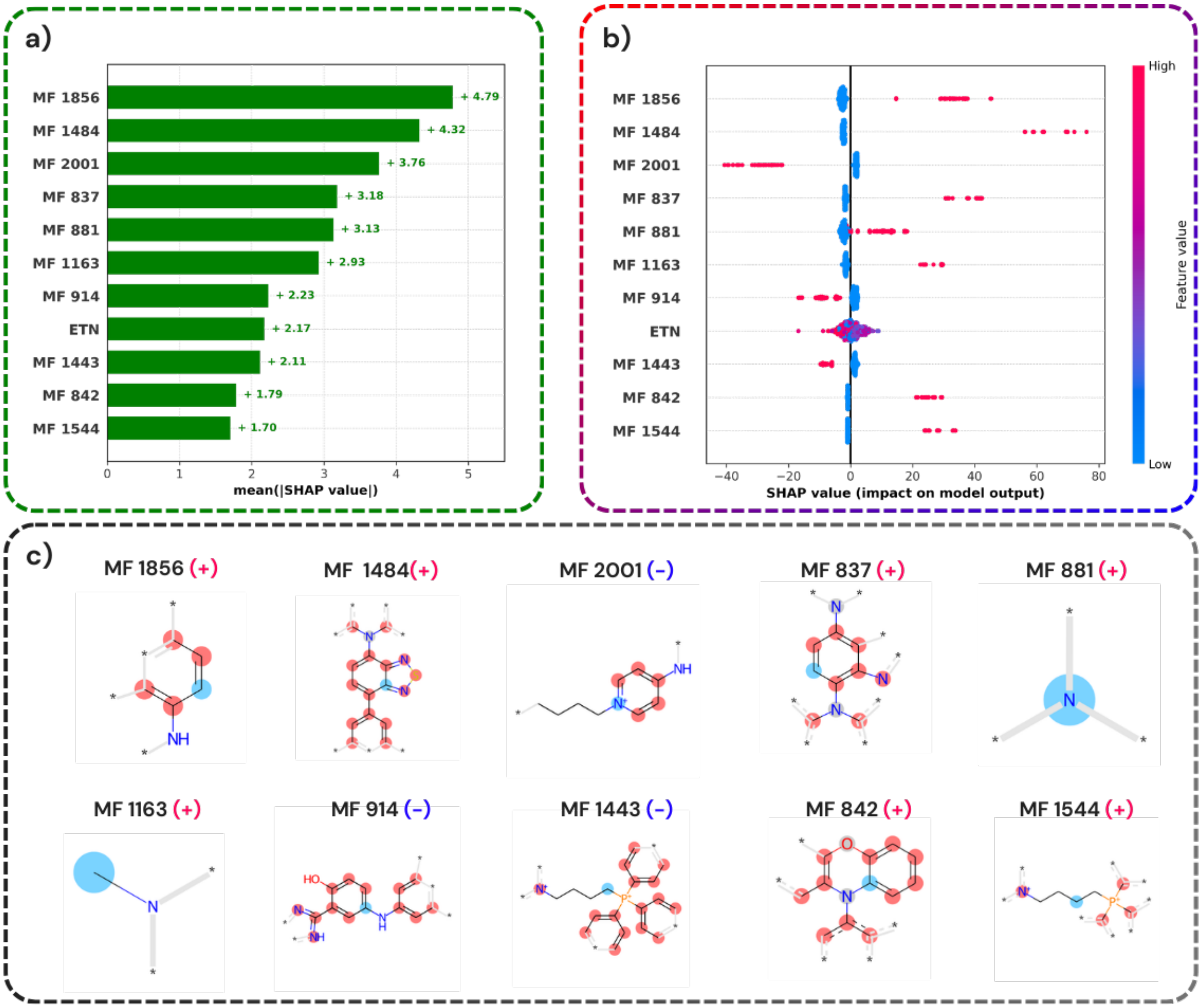


Figure S10: Visualization of SHAP analysis and molecular features influencing the predictive $\lambda_{max}^{abs}$ model. (a) Mean SHAP values of top molecular features (MFs) highlight their overall importance. (b) Bee swarm plot showing SHAP values for each molecular feature with color indicating feature magnitude (high: red, low: blue). (c) Molecular structures of selected features, classified as positively (+) or negatively (-) contributing to the model's output. For LGBM

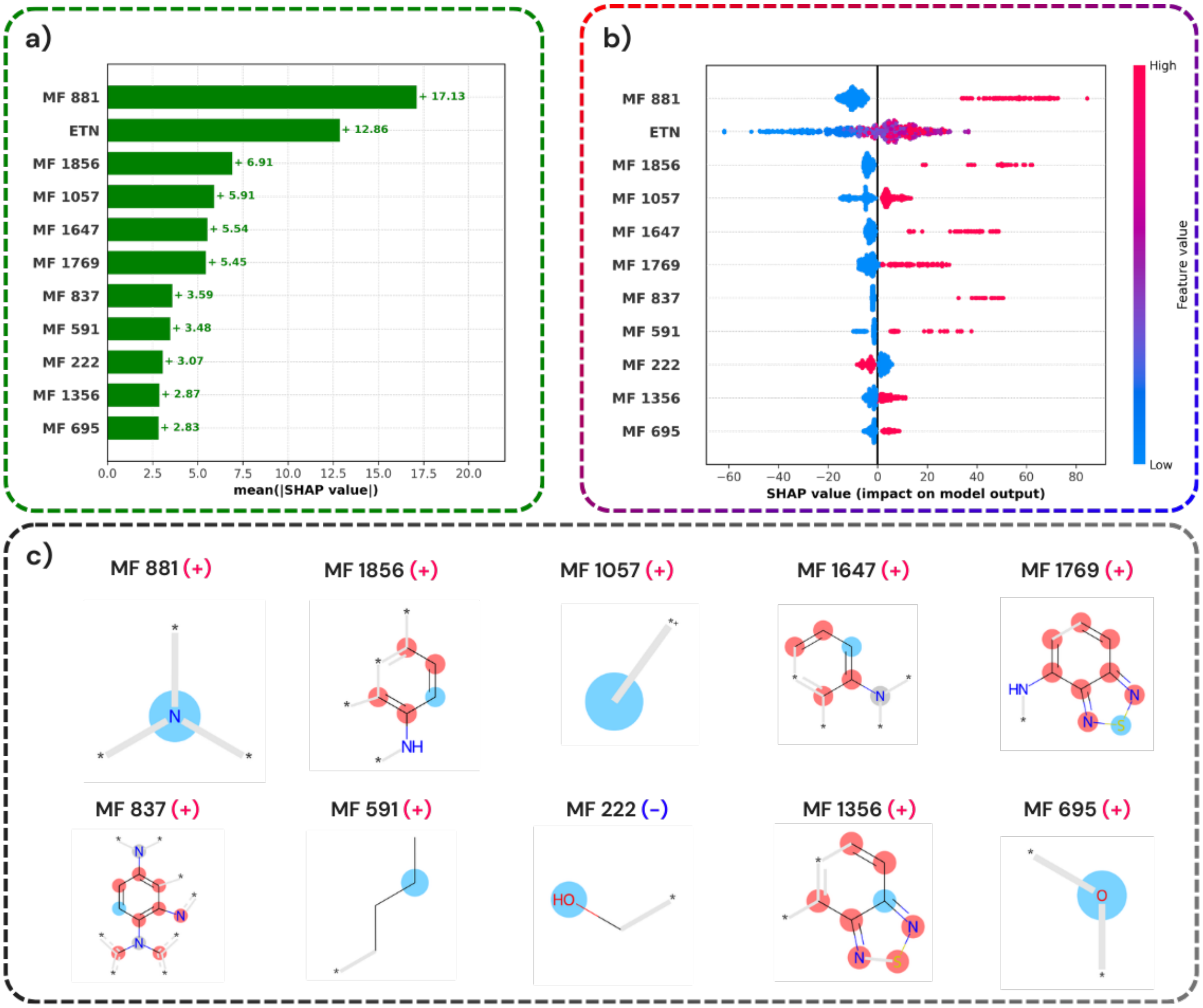


Figure S11: Visualization of SHAP analysis and molecular features influencing the predictive $\lambda_{max}^{em}$ model. (a) Mean SHAP values of top molecular features (MFs) highlight their overall importance. (b) Bee swarm plot showing SHAP values for each molecular feature with color indicating feature magnitude (high: red, low: blue). (c) Molecular structures of selected features, classified as positively (+) or negatively (-) contributing to the model's output. For LGBM

# References


(1) You, H.; Ma, Z.; Tang, Y.; Wang, Y.; Yan, J.; Ni, M.; Cen, K.; Huang, Q. Comparison of ANN (MLP), ANFIS, SVM, and RF models for the online classification of heating value of burning municipal solid waste in circulating fluidized bed incinerators. *Waste Management* **2017**, *68*, 186–197.

(2) Faceli, K. *Inteligência artificial: uma abordagem de aprendizado de máquina*; Grupo Gen - LTC, 2011.

(3) Welbl, J. Casting Random Forests as Artificial Neural Networks (and Profiting from It). Pattern Recognition. Cham, 2014; pp 765–771.

(4) Xing, J.; Wang, H.; Luo, K.; Wang, S.; Bai, Y.; Fan, J. Predictive single-step kinetic model of biomass devolatilization for CFD applications: A comparison study of empirical correlations (EC), artificial neural networks (ANN) and random forest (RF). *Renewable Energy* **2019**, *136*, 104–114.

(5) Chen, T.; Guestrin, C. XGBoost: A Scalable Tree Boosting System. Proceedings of the 22nd ACM SIGKDD International Conference on Knowledge Discovery and Data Mining. New York, NY, USA, 2016; p 785–794.

(6) Meng, Y.; Yang, N.; Qian, Z.; Zhang, G. What Makes an Online Review More Helpful: An Interpretation Framework Using XGBoost and SHAP Values. *Journal of Theoretical and Applied Electronic Commerce Research* **2021**, *16*, 466–490.

(7) Li, P.; Zhang, J.-S. A New Hybrid Method for China's Energy Supply Security Forecasting Based on ARIMA and XGBoost. *Energies* **2018**, *11*.

(8) Torlay, L.; Perrone-Bertolotti, M.; Thomas, E.; Baciu, M. Machine learning–XGBoost analysis of language networks to classify patients with epilepsy. *Brain informatics* **2017**, *4*, 159–169.

(9) Sanches-Neto, F. O.; Dias-Silva, J. R.; Keng Queiroz Junior, L. H.; Carvalho-Silva, V. H. "pySiRC": Machine Learning Combined with Molecular Fingerprints to Predict the Reaction Rate Constant of the Radical-Based Oxidation Processes of Aqueous Organic Contaminants. *Environmental Science & Technology* **2021**, *55*, 12437–12448, PMID: 34473479.

(10) Ke, G.; Meng, Q.; Finley, T.; Wang, T.; Chen, W.; Ma, W.; Ye, Q.; Liu, T.-Y. LightGBM: A Highly Efficient Gradient Boosting Decision Tree. Advances in Neural Information Processing Systems. 2017.

(11) Aziz, R. M.; Baluch, M. F.; Patel, S.; Ganie, A. H. LGBM: a machine learning approach for Ethereum fraud detection. *International Journal of Information Technology* **2022**, *14*, 3321–3331.
